# Impact of Inflammation on Ferritin, Hepcidin and the Management of Iron Deficiency Anemia in Chronic Kidney Disease

**DOI:** 10.3390/nu10091173

**Published:** 2018-08-27

**Authors:** Norishi Ueda, Kazuya Takasawa

**Affiliations:** 1 Department of Pediatrics, Public Central Hospital of Matto Ishikawa, 3-8 Kuramitsu, Hakusan, Ishikawa 924-8588, Japan; 2 Department of Internal Medicine, Public Central Hospital of Matto Ishikawa, 3-8 Kuramitsu, Hakusan, Ishikawa 924-8588, Japan; kazuya@takasawa.org; 3 Department of Internal Medicine, Public Tsurugi Hospital, Ishikawa 920-2134, Japan

**Keywords:** chronic kidney disease, ferritin, C-reactive protein, hepcidin, inflammation, iron deficiency anemia

## Abstract

Iron deficiency anemia (IDA) is a major problem in chronic kidney disease (CKD), causing increased mortality. Ferritin stores iron, representing iron status. Hepcidin binds to ferroportin, thereby inhibiting iron absorption/efflux. Inflammation in CKD increases ferritin and hepcidin independent of iron status, which reduce iron availability. While intravenous iron therapy (IIT) is superior to oral iron therapy (OIT) in CKD patients with inflammation, OIT is as effective as IIT in those without. Inflammation reduces predictive values of ferritin and hepcidin for iron status and responsiveness to iron therapy. Upper limit of ferritin to predict iron overload is higher in CKD patients with inflammation than in those without. However, magnetic resonance imaging studies show lower cutoff levels of serum ferritin to predict iron overload in dialysis patients with apparent inflammation than upper limit of ferritin proposed by international guidelines. Compared to CKD patients with inflammation, optimal ferritin levels for IDA are lower in those without, requiring reduced iron dose and leading to decreased mortality. The management of IDA should differ between CKD patients with and without inflammation and include minimization of inflammation. Further studies are needed to determine the impact of inflammation on ferritin, hepcidin and therapeutic strategy for IDA in CKD.

## 1. Introduction

Iron deficiency (ID) occurs in two major forms; absolute ID as defined by a decrease in the body iron stores and functional ID (FID), a disorder in which the total body iron stores are normal or increased but the iron supply to the bone marrow is inadequate [1]. ID anemia (IDA) is frequently associated with chronic inflammatory conditions, in which inflammation is pathogenically involved, including inflammatory bowel disease (IBD, e.g., ulcerative colitis, Crohn’s disease), chronic heart failure, chronic liver disease, obesity, rheumatoid arthritis (RA) and chronic kidney disease (CKD) [1]. IDA is a global public health problem affecting 7.2–13.96 per 1000 person-years and accounting for 800,000 deaths per year worldwide [2]. IDA also has an impact on mortality in children and adults with CKD [3,4]. Thus, appropriate management of IDA is crucial for improving quality of life (QOL) and mortality in CKD patients.

Iron is a component of heme proteins (hemoglobin; Hb and myoglobin), which carry or store oxygen and essential for the functioning of all organs. Iron is also present in heme enzymes, non-heme iron enzymes and iron-sulfur proteins that regulate various cell functions including electron transport in mitochondrial respiration, redox reactions and DNA synthesis [5]. However, excessive iron leaves a fraction of free iron (known as “labile” iron) and makes redox active, forming reactive oxygen species that cause oxidant stress. Because of no excretion system for iron from the body, iron homeostasis is tightly regulated via a network of proteins involved in the import, storage, export and transport of iron within the body.

There are two major molecules that regulate iron metabolism and iron availability for erythropoiesis. Ferritin binds iron as a ferric complex within a protein shell, in which iron fluxes in and out and functions as iron storage site and ferroxidase [5,6]. Thus, alteration of serum ferritin may be a determinant of mortality in adults on maintenance hemodialysis (HD) [7,8,9]. While iron supplementation reduced mortality in HD adults [9], excess iron therapy increased ferritin levels, leading to high mortality [7]. To date, optimal levels of serum ferritin during iron therapy for IDA remain to be determined in CKD patients. On the other hand, hepcidin-25 (referred to as hepcidin), a 25 amino acid peptide, is a major iron-regulatory hormone that binds to ferroportin (FPN) and inhibits iron export from enterocytes, hepatocytes and macrophages through the internalization and degradation of FPN, thereby regulating iron metabolism in various diseases, including CKD [10,11,12].

Inflammation as defined by the innate immune response to stimuli such as pathogens, cellular injury and metabolic stress [13] is part of the complex biologic response to tissue injury, infection, ischemia and autoimmune diseases. It is characterized by the acute-phase response including elevated inflammation markers such as C-reactive protein (CRP, >0.3 mg/dL) [14] and pro-inflammatory cytokines which promote CRP synthesis [15]. Inflammation is characteristic feature of CKD and caused by multiple factors of the toxic uremic milieu and the dialysis procedure itself. The interpretation of iron biomarkers is hindered by inflammation, which can directly affect the concentrations of most iron biomarkers [14], including ferritin and hepcidin [16,17,18]. Inflammation-mediated increase in hepcidin leads to iron trapping within the macrophages and hepatocytes, resulting in FID [19]. This leads to high association of inflammation with FID anemia (FIDA) in CKD patients [16,17,18], requiring higher dose of IV iron to maintain Hb targets [20]. Inflammation also induces hyporesponsiveness to iron therapy [20,21,22] and erythropoiesis-stimulating agents (ESA) in HD patients [21,23] by increasing ferritin and hepcidin. Conversely, aggressive intravenous iron therapy (IIT) may enhance inflammation in patients with end-stage renal disease (ESRD) [16], leading to further disturbance of iron metabolism. Thus, inflammation has an impact on the expression of ferritin and hepcidin as well as therapeutic strategy for the management of IDA in CKD patients.

The aim of this review is to provide an overview of clinical and experimental studies regarding a role of ferritin, hepcidin and inflammation in the regulation of IDA, efficacy of oral iron therapy (OIT) and IIT, predictive values of ferritin and hepcidin for the response to iron therapy, upper limit of ferritin levels to predict iron overload, optimal ferritin levels during iron therapy, complications and outcome in CKD patients. This review especially focuses on the impact of inflammation on these issues in CKD patients.

## 2. Ferritin Regulation by Iron Status and Inflammation

Ferritin has two isoforms; the heavy chain (FtH) and the light chain of ferritin (FtL). In contrast to FtH, FtL lacks detectable ferroxidase activity but can store more iron [6]. Ferritin sequesters iron in a nontoxic form, whereas the levels of free iron regulate cellular ferritin levels [6]. Cytoplasmic ferritin synthesis is stimulated by an increase of iron, while it is decreased by iron depletion. This process is mediated by the interaction between the two RNA-binding proteins (iron regulatory proteins 1 and 2; IRP1/2) and a region in the 5_untranslated region of FtH and FtL mRNA, termed the iron responsive element (IRE) that has a “stem-loop” structure. Binding of IRP1/2 to the IRE inhibits mRNA ferritin translation. IRP1 and IRP2 are differentially regulated, depending on iron status. When iron is abundant, IRP1 exists as a cytosolic aconitase, whereas under iron depletion, it assumes an open configuration associated with the loss of iron atoms in the iron-sulfur cluster and can bind to the IRE stem loop, thereby suppressing ferritin translation. IRP2 protein is abundant in iron depletion status but is rapidly degraded in iron excess. IRP1 and IRP2 have distinct tissue-specific roles [6].

Synthesis of FtH and FtL is activated by pro-inflammatory cytokines such as interleukin (IL)-1β and tumor necrosis factor (TNF)-α [6,24] via nuclear factor (NF)-κB pathway [25]. Interferon (INF)-γ and lipopolysaccharide (LPS) induce degradation of IRP2 in nitric oxide (NO)-dependent manner, leading to ferritin synthesis in macrophages [26]. IL-6 also enhances synthesis of FtH and FtL in hepatocytes [24]. FtH transcription is predominantly active in inflammatory conditions, whereas transcription of FtL can be induced only after exposure to very high concentration of iron [27]. Pro-inflammatory cytokines modulate the relative ratio of ferritin to body iron storage by increasing ferritin synthesis [27].

## 3. Hepcidin and Iron Metabolism

### 3.1. Hepcidin-Ferroporin Axis Regulates Iron Homeostasis

**Figure 1:**
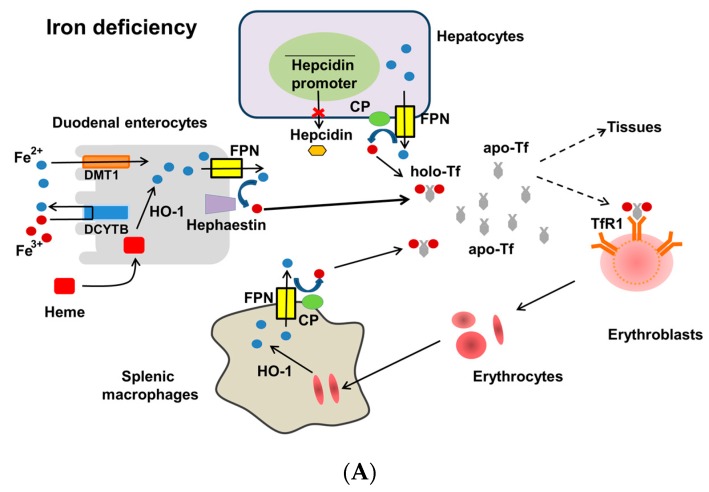

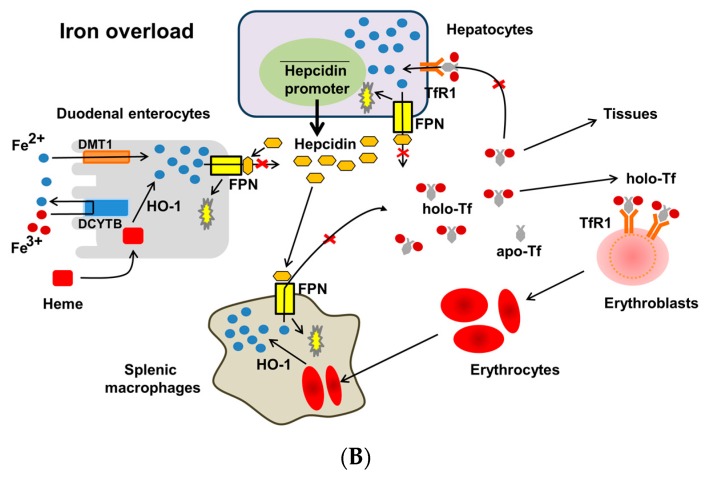
Regulation of systemic iron homeostasis. Divalent metal transporter 1 (DMT1) at the apical membrane of enterocytes takes up Fe^2+^ from the lumen after duodenal cytochrome b (DCYTB) reduces dietary Fe^3+^ to Fe^2+^. Ferroportin (FPN) at the basolateral membrane exports Fe^2+^ into the circulation. FPN cooperates with hephaestin that oxidizes Fe^2+^ to Fe^3+^. In hepatocytes and macrophages, Fe^2+^ is oxidized by a ferroxidase, ceruloplasmin (CP). Diferric (Fe^3+^_2_) transferrin (holo-Tf) supplies iron to all cells and tissues through binding to Tf receptor 1 (TfR1) and endocytosis. Erythrocytes are phagocytized by macrophages. Hemoglobin-derived heme in enterocytes and macrophages is catabolized by heme oxygenase-1 (HO-1). After sensing iron, hepatocytes produce and release hepcidin. In iron deficiency (**A**), low hepcidin facilitates iron export by FPN into the circulation. In iron overload (**B**), high hepcidin binds to FPN and inhibits iron export from enterocytes, hepatocytes and macrophages by triggering internalization and degradation of FPN, leading to reduction of iron storage. Dashed line indicates less iron supply. x: inhibition. Figures adapted from [12].

Dietary iron containing heme and non-heme iron is absorbed by the divalent metal transporter (DMT1) located at the apical membrane of duodenal enterocytes [10,11,12] (Figure 1), which transports only Fe^2+^ but most dietary iron is Fe^3+^. Thus, Fe^3+^ should be reduced to Fe^2+^ before iron transport by DMT1. This reduction step is mediated by the ferrireductase duodenal cytochrome b (DCYTB) at the apical membrane of duodenal enterocytes. Hepcidin binds to FPN and triggers its internalization, ubiquitination and subsequent lysosomal degradation, leading to inhibition of iron export by FPN from enterocytes, hepatocytes and macrophages into the circulation. Heme absorbed by the enterocytes is degraded by heme oxygenase-1 (HO-1) and the liberated iron is processed in a similar manner as the absorbed inorganic iron. The export of enterocytic iron by FPN requires hephaestin, a multicopper oxidase homologous to ceruloplasmin (CP), which oxidizes Fe^2+^ to Fe^3+^ for loading onto transferrin (Tf) [10]. Once exported by FPN into the circulation, Fe^2+^ is oxidized into Fe^3+^ by a ferrioxidase CP in hepatocytes and macrophages. Iron-loaded (diferric) transferrin (Tf-Fe_2_, holo-Tf) is transported to other cells or tissues for iron metabolism or storage [10,11,12].

Hepatocytes sense iron stores and regulate hepcidin promotor activity, thereby releasing hepcidin accordingly. In ID (Figure 1A), hepcidin is low and thus iron is released by FPN into the circulation from enterocytes, hepatocytes and macrophages, facilitating iron availability for erythropoiesis [10,11,12]. Under iron overload (Figure 1B), increased hepcidin in hepatocytes is released [10,11,12]. Increased hepcidin binds to FPN and inhibits FPN-mediated iron export into the circulation to reduce Tf saturation (TSAT), leading to subsequent inhibition of duodenal iron absorption.

### 3.2. Hepcidin Regulation by Iron Status

**Figure 2:**
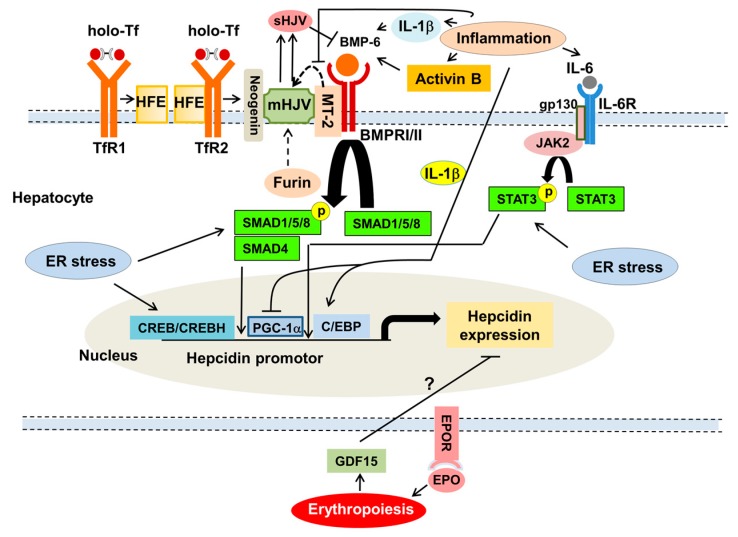
Regulation of hepcidin by iron status and inflammation. Under high iron conditions, increased holo-Tf induces bone morphogenetic protein (BMP)-6 in non-parenchymal cells in the liver, disrupts hereditary hemochromatosis protein (HFE)-transferrin receptor (TfR)1 interaction to promote HFE-TfR2 interaction and its association with membrane-anchored hemojuvelin (mHJV), forming a complex of HFE/TfR2/BMP-6/BMPR/HJV/neogenin, which is dispensable for hepcidin transcription via BMP/small mothers against decapentaplegic (SMAD) signaling. In iron overload, furin, which cleaves mHJV, is downregulated, thereby increasing hepcidin. Low iron conditions increase matriptase (MT)-2 and furin, which cleaves mHJV, reduces BMP-6 production and facilitates the HFE-TfR1 interaction, leading to inhibition of BMP/SMAD-dependent hepcidin transcription. Pro-inflammatory cytokines such as interleukin (IL)-1β and IL-6 stimulate hepcidin expression via the Janus kinase (JAK)/signal transducers and activators of transcription (STAT)3 signaling. Inflammation induces other cytokine, activin B, which stimulates BMP/SMAD signaling, synergically with IL-6 and STAT3 signaling, leading to hepcidin expression. Endoplasmic reticulum (ER) stress associated with inflammation increases hepcidin via SMAD1/5/8 and cyclic-AMP-responsive-element-binding protein (CREB)H that binds and activates hepcidin promoter activity. Inflammation increases hepcidin by inhibiting MT-2 via decreased STAT5 and peroxisome proliferator-activated receptor γ coactivator (PGC)-1α which antagonizes lipopolysaccharide-induced hepcidin transcription via the interaction with hepatocyte nuclear factor 4α. Inflammation-induced IL-1β enhances hepcidin transcription by inducing CCAT enhancer-binding protein (C/EBP)δ. Hepcidin translation is mediated indirectly through erythropoietin (EPO)/EPOR-induced erythropoiesis and possibly growth differentiation factor (GDF)15. gp130: glycoprotein 130. Dashed line: cleavage, →: activation, ├: inhibition.

After sensing iron, bone morphogenetic protein (BMP)-6 is produced in the liver [28] and increases hepcidin via BMP/small mothers against decapentaplegic (SMAD) proteins-mediated signaling pathway [28] (Figure 2). Holo-Tf displaces the interaction of hereditary hemochromatosis protein (HFE)-TfR1 and stabilizes the association of HFE-TfR2 with membrane-anchored hemojuvelin (mHJV), forming a complex of HFE/TfR2/HJV which is dispensable for hepcidin transcription [10,11,28]. mHJV exclusively expressed in hepatocytes acts as a co-receptor for BMP-6, leading to activation of BMP/SMAD-mediated hepcidin transcription [28]. Neogenin acts as a scaffold to facilitate assembly of HJV/BMP/BMP receptor (BMPR) complex [29] and maintains proper mHJV function [28]. Matriptase (MT)-2 (also known as TMPRSS6) functions as a negative regulator of hepcidin-related BMP/SMAD signaling by cleaving mHJV into soluble HJV (sHJV) [11].

ID reduces BMP-6, leading to inhibition of BMP/SMAD-dependent hepcidin transcription. Decreased holo-Tf in ID destabilizes the TfR2-HFE interaction, thereby inhibiting hepcidin transcription [28]. Decreased holo-Tf and non-Tf-bound iron increase sHJV, leading to inhibition of hepcidin, while increased holo-Tf and non-Tf-bound iron have the opposite effect [28]. ID stabilizes MT-2, thereby suppressing hepcidin [28,30]. Furin, which cleaves mHJV into sHJV, is upregulated by ID and inhibits hepcidin [31]. In iron overload, increased holo-Tf stabilizes the TfR2-HFE interaction [10,11,28] and decreases furin [32], thereby increasing hepcidin translation.

### 3.3. Inflammation Increases Hepcidin Expression

Pro-inflammatory cytokines are increased in CKD [17,18]. Pro-inflammatory cytokines such as IL-1β and IL-6 stimulate hepcidin expression via the Janus kinase (JAK)/signal transducer and activator of transcription 3 (STAT3) pathways [10,11,12,28] (Figure 2). Inflammation induces other cytokine activin B which stimulates BMP-6/SMAD pathway synergically with IL-6 and STAT3, leading to hepcidin expression [11]. Endoplasmic reticulum (ER) stress associated with inflammation increases hepcidin by activating SMAD1/5/8 [33], IL-6-dependent phosphorylated STAT3 [34] and ER stress-activated transcription factor, cyclic AMP response element–binding protein H (CREBH), which bind and activate hepcidin promoter activity [35]. Inflammation inhibits MT-2 by suppressing STAT5 [36] and peroxisome proliferator-activated receptor γ coactivator-1α (PGC-1α) which antagonizes LPS-induced hepcidin transcription via the interaction with hepatocyte nuclear factor 4α [37], leading to activation of hepcidin translation. Inflammation-induced IL-1β also activates hepcidin expression by inducing CCAAT enhancer-binding protein (C/EBP)δ in hepatocytes [38].

## 4. Biomarkers of Iron Status and Inflammation in CKD

A number of biomarkers of iron status have been used in a clinical setting. However, traditional biochemical iron parameters such as serum iron, Tf and serum ferritin are influenced by inflammation [14], making them less suitable indicators. Due to confounding effects of the acute-phase response on the interpretation of most iron indicators, the assessment of iron status is challenging when concomitant inflammation is present. In fact, serum levels of ferritin were positively correlated with CRP, a measure of inflammation, in children and adults on HD [16,39,40]. Under minor inflammation, serum ferritin appears to be a most reliable biomarker of total body iron stores and ID is diagnosed below the cutoff serum ferritin levels of <15 ng/mL in individuals older than 5 years [1]. However, serum ferritin levels of 50 ng/mL or higher could still be ID when apparent inflammation is present [1]. Thus, the interpretation of serum ferritin, an acute phase reactant, is complicated by concomitant inflammation [1,14,41].

As negative acute phase reactants, concentrations of serum iron and Tf, an iron transport protein, are decreased in response to inflammation [14,41,42,43]. Low TSAT (<20%) with low serum ferritin is diagnostic of IDA [1,44]. Low TSAT combined with normal or elevated serum ferritin is diagnostic of FIDA [44]. As discussed later, many international guidelines for the management of IDA in CKD use the combination of low serum ferritin and TSAT for diagnosis of IDA. TSAT represents the percentage of binding sites on all Tf molecules occupied with iron molecules and is calculated as the ratio of serum iron to Tf or serum iron to total iron-binding capacity (TIBC) which indicates the maximum amount of iron necessary to saturate all available transferrin iron-binding sites [1,44]. TIBC is a negative acute-phase reactant and reduction in TIBC induced by inflammation leads to higher TSAT levels independent of iron status [41]. Thus, reliability of TSAT as a measure of iron status is reduced by inflammation associated with CKD. Soluble TfR (sTfR), which is produced by proteolysis of the membrane TfR, is increased in HD patients with ID and inversely correlated with the amount of iron available for erythropoiesis [44]. As a marker of iron status, sTfR is not an acute-phase reactant and less influenced by inflammation than serum ferritin is, while it is increased in individuals with general inflammation [14]. In addition, sTfR appears to represent erythropoietic activity more than iron-restricted erythropoiesis in CKD patients receiving ESA [44]. Thus, the interpretation of sTfR is also confounded by the use of ESA [1,41]. Thus, it is an inferior marker to cellular measures such as the content of Hb in reticulocytes (CHr) or the percentage of hypochromic red blood cells (%Hypo) [41,44]. Other limitations of sTfR measurement include cost for the measurement and lack of standard cutoff and widespread availability [1,41]. The interpretation of total body iron (TBI), the log ratio of sTfR to serum ferritin, is also complicated by the same confounding factors as for serum ferritin and sTfR concentrations [14].

Other laboratory markers of iron status include CHr and %Hypo. Both iron biomarkers are influenced by inflammation [45,46]. CHr is inversely related to log CRP [45] and there is a positive correlation between %Hypo and CRP [46]. CHr is a very early index of iron available for erythropoiesis within 3–4 days [1,41,44]. The CHr of <27.2 pg is diagnostic of ID, while false normal values are frequently encountered [1]. The measurement of %Hypo as a proportion of hypochromic cells defined as erythrocytes with mean cellular Hb concentration less than 28 g/dL in total red blood cells is the most sensitive marker of ID in CKD (cutoff, <6%) [1] and FID [41]. However, it is not suitable for the assessment of short-term changes in iron status [1,41,44]. Furthermore, a fresh blood sample is needed and automated analyzers are not widely available in clinical setting. Currently, none of the measurements are adequate and accurate indicators of iron status, especially when concomitant inflammation is present.

It is controversial about whether hepcidin is a reliable biomarker of iron status in CKD patients. Serum hepcidin has been shown to be positively correlated with serum ferritin, percent iron saturation, CRP and sTfR and negatively with glomerular filtration rate (GFR) in CKD patients [47]. As GFR decreased, serum hepcidin levels were increased in non-dialysis (ND)-CKD patients [48]. Dialysis can remove hepcidin [49] and inflammation increases hepcidin like ferritin [18,48,50]. A significant intra-individual variability of hepcidin was dependent on short-term fluctuations in the inflammatory condition [50]. Thus, short-term measurement of serum hepcidin should not be used as a biomarker of iron status in HD patients [50]. Interpretation of the data for serum hepcidin should be with caution due to the confounding factors as described. However, hepcidin could be a good biomarker of iron status in CKD patients in the absence of apparent inflammation.

## 5. IDA in CKD

### 5.1. Impact of Inflammation on Diagnosis of IDA

The cutoff levels of Hb for diagnosis of anemia depend on age, sex and pregnancy [1] (Table 1). In general, low serum ferritin (<15 ng/mL) and TSAT (<16%) are used for diagnosis of IDA in individuals without inflammatory conditions [1]. However, IDA could be diagnosed based on higher cutoff levels of serum ferritin and TSAT in the presence of chronic inflammatory condition such as CKD [1]. Table 2 summarizes diagnostic criteria for IDA in ND-CKD and HD patients used by international guidelines for the management of IDA in CKD patients [51,52,53,54,55,56,57,58,59,60]. Some guidelines in Canada, the US [51,52,54] and Taiwan [57] recommend higher cutoff levels of serum ferritin ≤200–500 ng/mL and TSAT ≤30% [54,57] for diagnosis of IDA and initiation of iron supplementation in ND-CKD and HD patients than those (serum ferritin <100 ng/mL and TSAT <20%) recommended by the Japanese Society for Dialysis Therapy (JSDT) guidelines [53] and other guidelines from Europe and Australia [55,56,58,59,60]. The Japanese nationwide study showed low cutoff levels of serum ferritin (<50–100 ng/mL) and TSAT (<20%) to diagnose IDA in HD patients with low CRP (median, 1.0 mg/mL) [61]. The reason for the difference in the criteria for diagnosis of IDA and the initiation of iron therapy in CKD patients between Japan and some Western countries is unclear. However, it may be in part due to lower prevalence of inflammation associated with HD patients in Japan than those in Western countries [15,61,62]. While the specific biologic basis underlying differences by race and ethnicity is unclear [62], one of the reasons may be higher prevalence of arteriovenous (AV) fistula and lower prevalence of. catheter use and AV graft in Japan [15,63,64] that are associated with higher levels of CRP [65] as compared to Western countries. Nonetheless, the impact of inflammation could increase the cutoff levels of serum ferritin and TSAT for diagnosis of IDA to initiate iron therapy in CKD patients.

### 5.2. IDA, Inflammation and Clinical Outcome in CKD

IDA and FIDA are frequently associated with chronic inflammation, including CKD. IDA occurs in 42.0% in children with CKD [66] and 1.2–13.9% in those without [67,68] as well as in 24.0–85.0% of adults with CKD [69,70] and 1.0–4.5% of those without [67,71], suggesting that IDA is more frequently associated with CKD patients than general population. IDA is more prevalent in both girls and women than boys and men and in patients with advanced CKD than in those with low grade CKD [66,69]. FIDA occurs in 12.0–21.4% of ND-CKD adults [18,48] and 23.0–42.9% of HD adults [17,72] although prevalence of FIDA remains unknown in CKD children and general population. Thus, both IDA and FIDA more frequently occur as CKD advances.

IDA increases a risk of hospitalization [73] and mortality, including all-cause and cardiovascular-related mortality in ND-CKD [4,73,74] and HD adults [75,76]. Several short-term studies showed that Hb < 10 g/dL was a risk factor of mortality in ND-CKD [77] and HD adults [78,79]. The long-term studies showed that Hb levels of <10–12 g/dL [80,81,82] were a risk factor of mortality in HD adults. On the other hand, in HD children, Hb <12 g/dL was associated with increased risk of hospitalization and mortality [83]. These data suggest that the levels of Hb of < 12 g/dL are a risk factor of mortality in both children and adults with ND-CKD and HD. Additionally, longer time required to reach the tHb level was associated with higher risk of hospitalization and mortality in HD adults [84]. Thus, early intervention with iron therapy for IDA can improve QOL and mortality in CKD patients.

Little is known whether inflammation affects the cutoff levels of ferritin deficiency as a risk factor of mortality in CKD patients. The study from Europe reported that in HD adults, mortality rate was 13.8/100 patient-years and that serum ferritin levels of <100 ng/mL were a risk factor of cardiovascular-related mortality in HD patients with positive CRP [76]. However, the Japanese study showed that low serum ferritin levels of either <30 ng/mL or <50 ng/mL were associated with a significant risk of mortality in Japanese HD patients with normal CRP receiving IIT and ESA [8,85]. These data suggest that low levels of serum ferritin are a risk factor of mortality in CKD patients, whereas the cutoff levels of ferritin deficiency to predict mortality is lower in HD patients without than those with apparent inflammation. 

In addition to low ferritin, low serum iron (<45.3 μg/dL) [86], TSAT (≤20%) [74,87,88] and TIBC [75] have been shown to be risk factors of hospitalization and mortality in ND-CKD and HD adults. IDA induces resistance to ESA [88] which aggravates renal anemia, leading to worse outcome in HD patients [89]. These data suggest that IDA is a significant risk factor of mortality in CKD patients. As discussed earlier, inflammation affects these indicators of iron status, especially the cutoff levels of serum ferritin deficiency to predict mortality in CKD patients.

### 5.3. Inflammation, Increased Serum Ferritin and Mortality in CKD

The management of IDA with iron supplementation is crucial for better outcome in CKD patients. However, aggressive iron therapy causes iatrogenic iron overload, leading to inflammation and increasing a risk of mortality. In support of this hypothesis, inflammation and hyperferritinemia (>500 ng/mL) were associated with cardiovascular and all-cause mortality in HD adults [90]. In addition, in dialysis children and adolescents with iron overload, ferritin levels showed a positive correlation with inflammation markers (CRP and IL-6) and left ventricular mass (LVM) [39].

It is controversial whether iron overload is associated with a risk of infection or infection-related mortality [91]. High serum ferritin has been shown to be an independent risk factor of infection-related mortality in HD patients [7,92,93,94,95]. Bolus dosing of ferric gluconate could increase a risk of infection-related mortality and hospitalization in HD patients with catheter [96]. However, no relation has been reported between ferritin and a risk of infection-related mortality in HD patients [97].

To reduce a risk of iron overload-mediated toxicity, international guidelines for the management of IDA in CKD recommend that upper limit of serum ferritin and TSAT should be maintained at <500–800 ng/mL [51,52,53,54,55,56,57,58,59,60] and <30–50% [53,55,57], respectively (Table 2). Other study groups in Europe and the US recommend that serum ferritin should be maintained at 400–600 ng/mL [98] and 200–1200 ng/mL [99] in HD patients. The percentage of US facilities with an upper ferritin target of 1200 ng/mL increased from 20% to 40% from 2010 to 2011 and more than 90% facilities had an upper ferritin target of 800 ng/mL in 2014 [100]. Upper ferritin targets of 500 ng/mL remained common in Europe and no European facility had upper ferritin targets of 1200 ng/mL in 2014. In Japan, upper ferritin targets were lower, with most facilities targeting upper limits of 300 ng/mL [100]. In this study, the data for CRP were negative in Japanese facilities although data for CRP in US facilities are not available. These guidelines and the studies described here except for those from Japan are likely to be based on the data obtained from inflamed CKD patients as suggested by other studies [15,61,62]. Thus, inflammation significantly affects upper serum ferritin targets to avoid iron overload for the management of IDA in CKD patients. In support of this hypothesis, in the setting of concomitant inflammation, serum ferritin levels of ≥500–800 ng/mL are found to be predictive of high mortality in HD patients of Europe [9,101] and Taiwan [92] and USA [95]. High CRP levels alone may predict high mortality in HD patients [62,102]. Taken together, these data suggest that iron overload as reflected by high serum ferritin and concomitant inflammation independently or synergically can increase a risk of mortality in CKD patients.

By contrast, in the absence of inflammation, even slight increase in serum ferritin (≥200 ng/mL) is associated with a risk of mortality in Japanese HD patients [85]. Thus, the cutoff levels of serum ferritin to predict iron overload and a risk of mortality are significantly lower in HD patients without than in those with apparent inflammation. Since inflammation increases ferritin independent of iron status, upper serum ferritin targets to predict iron overload and mortality may be lower than >500–800 ng/mL in HD patients with apparent inflammation. Further studies would be necessary to determine what are true levels of upper serum ferritin targets to predict iron overload in CKD patients when concomitant inflammation is present.

## 6. Impact of Inflammation on Therapeutic Strategy with Iron Supplementation in CKD

Iron supplementation has been used in 57.3–78.0% of dialysis children [103,104,105] and its use is less prevalent in those not receiving than in those receiving ESA [104,105]. Thus, iron therapy is likely to be more frequently used in severe anemic CKD children receiving ESA. There is a similar trend in CKD adults; iron therapy has been used in 48.1–56.3% of ND-CKD [4,106], 68.0% of peritoneal dialysis (PD) and 76.0–84.4% of HD adults [104,107]. The use of IIT has recently been increased to >70% of HD adults [108] and serum ferritin targets and IV iron doses have been increased in HD adults [108,109]. Maintenance IIT can improve the response to ESA with a reduction of ESA dose and early survival in ND-CKD [73] and HD adults [110]. However, Hb levels of >13 g/dL following IIT is associated with worse outcome in CKD patients [111]. Thus, the target Hb (tHb) of 10–13 g/dL is recommended for the management of IDA in CKD patients [112,113,114]. We herein discuss the impact of inflammation on some therapeutic issues to maintain tHb for the management of IDA in CKD patients.

### 6.1. Inflammation and the Response to Iron Supplementation in CKD

IIT is generally superior to OIT to improve IDA in children and adults with CKD [115,116], while some patients fail to respond to iron therapy. In support of this, Qunibi et al. showed that the tHb levels were more maintained in ND-CKD patients (60.4%) after IV ferric carboxymaltose than in those (34.7%) receiving OIT [117]. One of the reasons accounting for superiority of ITT over OIT to maintain tHb in CKD patients may be difference in the efficacy of IIT and OIT in CKD patients in the presence of concomitant inflammation that increases ferritin and hepcidin. Table 3 summarizes data regarding the impact of inflammation on the effect of OIT versus IIT for IDA in CKD patients, in whom the data for CRP are available. The efficacy of IIT is generally better than OIT for the management of IDA in inflamed ND-CKD patients with positive CRP [98,118,119,120,121,122]. Stoves et al. showed no significant difference in the Hb levels at the end of OIT and IIT in ND-CKD adults with positive CRP, while the levels of Hb achieved tended to be higher in the IIT group than those in the OIT group [123]. In contrast, in HD patients with normal CRP, OIT and low dose of IIT is as effective as the standard IIT [124,125,126]. HD adults responding to OIT showed lower CRP levels (mean, 0.8 mg/dL) compared to those in the OIT-non-responders (mean, 5.6 mg/dL) [122]. These data suggest that superiority of IIT over OIT for the management of IDA in CKD patients [115,116,117] is true in the presence of apparent inflammation but not under minor inflammation and that inflammation may induce hyporesponsiveness to iron therapy, especially to OIT. The HD patients with high CRP levels had lower intestinal iron absorption [127] probably due to inflammation-induced increase in ferritin and hepcidin that block iron absorption [128]. Thus, inflammation makes OIT less effective to maintain tHb in CKD patients. Due to an alteration of ferritin and hepcidin by inflammation, higher dose of IV iron may be required to maintain tHb for IDA in CKD patients with concomitant inflammation.

In support of this hypothesis, an inflammation marker CRP was inversely correlated with Hb in HD patients [129,130,131]. In HD patients receiving IIT and ESA, below tHb was associated with high CRP [132]. In addition, high CRP with low CHr and TSAT led to a lack of response to further increment of IV iron dose in HD patients [21]. Serum hepcidin and inflammation markers (CRP, IL-6 and TNF-α) were more increased as CKD advanced [18,133,134,135], in which more severe IDA occurs. Furthermore, serum hepcidin was positively correlated with inflammation markers (CRP, IL-6 and TNF-α) and ferritin but inversely correlated with TIBC, Hb, Ht and GFR [50,134,135]. In contrast, relatively low CRP (≤6.5 mg/dL) was associated with more achievement of tHb and if CRP increased by 1 mg/dL, possibility to maintain tHb was reduced by 7.5% in HD patients [136]. These data suggest that inflammation is a confounding factor for maintenance of tHb probably due to increased ferritin and hepcidin which inhibit iron absorption and efflux, leading to reduced iron bioavailability for erythropoiesis.

Other supportive findings for an impact of inflammation on the response to iron therapy come from the case of FIDA in CKD patients accompanied by inflammation [18]. Serum inflammation markers (CRP and IL-6), ferritin and hepcidin were increased in HD patients with FIDA [17,18]. Iron absorption was reduced in HD patients with FIDA, in particular in those with high CRP [127]. FIDA in HD adults could be managed by IIT [20,137] but not OIT [137]. However, the overall response rate to IIT was only 46.3% in HD adults with FIDA, while IIT produced a significant but only small increase in Hb (mean, 0.54 g/dL) [20]. In addition, in CKD patients, IIT increased serum hepcidin levels, which in turn exacerbated FIDA, requiring higher doses of IV iron to maintain tHb [128]. Taken together, these clinical observations indicate that as CKD advances, inflammation worsens and increases ferritin and hepcidin, leading to inhibition of iron absorption and efflux and subsequent hyporesponsiveness to iron therapy. As a result, higher dose of IV iron may be required for the management of IDA in CKD patients with apparent inflammation.

### 6.2. Does Autoimmune Disease and Inflammatory Disorders That Lead to CKD/ESRD Affect the Response to Iron Therapy?

If inflammation could induce hyporesponsiveness to iron therapy by increasing ferritin and hepcidin, question arises as to whether inflammation associated with autoimmune and inflammatory disorders coexisting or underlying CKD has similar effects. The following clinical observations may answer this question. First, IDA and FIDA frequently occur in autoimmune and inflammatory disorders such as systemic lupus erythematosus (SLE) [138], RA [139,140] and IBD [141,142]. These disorders are frequently associated with CKD, leading to ESRD [143,144]. In addition, serum levels of ferritin and inflammation markers (CRP and IL-6) were higher in patients with active than in those with inactive SLE and controls [145,146,147] and IL-6 inversely correlated with Hb between active and inactive SLE [147,148]. Serum ferritin was positively correlated with SLE disease activity index, anti-dsDNA, IFN-γ, IL-6, proteinuria and renal dysfunction and negatively correlated with C3, C4 and Hb [140,145,146,147]. Thus, inflammation increases ferritin and worsens anemia in SLE [148]. Furthermore, urine levels of hepcidin were higher in adults with lupus nephritis (LN) than in lupus patients without LN and controls [149] and in LN patients with severe renal histology compared to those with mild lesions [149,150]. The kidney biopsy specimens from LN patients revealed infiltration of interstitial leukocytes expressing hepcidin [150]. Thus, hepcidin levels increase as LN advances, leading to further inhibition of iron absorption and efflux which may worsen anemia.

As other autoimmune disease, in active RA patients, serum levels of ferritin and hepcidin were significantly higher than those in inactive patients [140] and positively correlated with CRP and negatively with Ht or Hb [140,151]. In addition, serum levels of hepcidin were increased in IBD patients with iron malabsorption and positively correlated with serum levels of ferritin and CRP [152]. Furthermore, patients with IBD having higher levels of CRP achieved a lower Hb response with OIT than those with lower levels of CRP [153]. Thus, IIT has been more efficacious than OIT in patients with RA, SLE [154,155] and IBD [142]. Taken together, these data suggest that inflammation in autoimmune disorders and IBD leading to CKD/ESRD may have an impact on the expression of ferritin and hepcidin, the response to iron therapy and mode of iron therapy as well. Future studies would be necessary to address whether inflammation in autoimmune disease and IBD coexisting or underlying CKD has similar impact on the response to iron therapy as in CKD patients.

### 6.3. Inflammation May Reduce Predictive Values of Ferritin and Hepcidin for the Response to Iron Therapy

It is controversial whether baseline serum ferritin is predictive of the response to iron therapy in CKD patients with concomitant inflammation. Inflammation was associated with high serum ferritin (≥500 ng/mL) and conversely, this ferritin level and low TSAT (<25%) had higher odds ratio for serum CRP levels of ≥10 mg/dL in HD adults [156]. In addition, the levels of CRP were positively correlated with those of serum ferritin in HD patients [157] and higher levels of CRP and ferritin were associated with lower Hb levels in ESRD patients [129]. These data suggest that under concomitant inflammation, ferritin is not a predictor of the response to iron therapy. In support of this, Musanovic et al. reported that the predictive values of CRP to achieve tHb following IIT was ≤6.5 mg/dL and that serum ferritin levels failed to predict the response to IIT in highly inflamed HD patients with higher CRP levels [136]. In addition, in HD patients with high levels of serum ferritin (500–1200 ng/mL) and CRP (>20 mg/dL) and low TSAT (<25%), none of the iron indices (ferritin, CHr, TSAT or sTfR) was predictive of the response to IIT, while lower CRP levels of ≤14.0 mg/dL were predictive of the response to IIT [22]. Furthermore, in HD patients with moderately increased levels of serum ferritin (mean, 146 ng/mL) and CRP (mean, 4.1 mg/dL), %Hypo and CHr but neither ferritin nor TSAT were predictors of the response to IIT in HD patients [158]. These data suggest that the values of CRP but not ferritin may be predictive of the response to iron therapy in inflamed CKD patients.

In contrast, Macdougall et al. showed that in ND-CKD adults with relatively low CRP levels (mean, 4.5 mg/dL), the response rate to OIT was very limited (21.6%), whereas both low baseline ferritin and CRP may be predictive of the response to OIT [121]. In addition, serum ferritin levels were higher in HD patients with minor inflammation not responding to IIT than in those responding to IIT and OIT as described in our previous study [124]. Thus, basal ferritin may be predictive of the response to IIT in CKD patients with minor inflammation.

Regarding an impact of inflammation on predictive value of hepcidin for the response to iron therapy, serum hepcidin and prohepcidin were positively correlated with ferritin and CRP in HD patients [47,50,134,158]. Prohepcidin was negatively correlated with Hb and Ht in ND-CKD and dialysis patients with positive CRP and hyperferritinmia [134]. No correlation was found between hepcidin and TSAT and Hb in inflamed ND-CKD [159] and HD patients with FIDA [18]. In addition, serum levels of hepcidin failed to predict the response to IIT in adults on HD receiving ESA, in whom the levels of CRP and serum ferritin were relatively high [158] or in ND-CKD adults not receiving ESA with relatively high levels of CRP (mean, 6.7 mg/dL) [159]. The latter study also showed a correlation between hepcidin and ferritin levels at baseline and at the study endpoint, whereas an association between hepcidin and ferritin was plateaued at higher ferritin levels [159]. These data suggest that hepcidin is not a good predictor of the response to iron therapy in inflamed CKD patients.

Under minor inflammation, correlation between iron indicators (ferritin and TSAT) is more robust in HD patients [160]. In this condition, there was a negative association between hepcidin and Hb in ND-CKD adults with sufficient iron stores (serum ferritin ≥ 91 ng/mL) and in contrast, a positive association in those with ID (serum ferritin < 91 ng/mL) [161]. This finding suggests that under minor inflammation, the values of hepcidin to predict the response to iron therapy in CKD patients may be dependent on the levels of ferritin. In support of this hypothesis, hepcidin was positively correlated with ferritin (mean, 50.6 ng/mL) and TSAT in HD adults with minor inflammation [125]. In addition, low levels of serum hepcidin and ferritin with normal or slightly positive CRP levels were able to predict a greater response to OIT or IIT in ND-CKD [162] and HD patients [113,124]. Furthermore, lower levels of hepcidin and higher sTfR and sTfR/hepcidin ratio were predictive of the response to IIT in ND-CKD patients with relatively low CRP (mean, 3.8 mg/dL) [163]. Taken together, under minor inflammation in which correlation between hepcidin, ferritin and Hb is more robust, hepcidin may be predictive of the response to iron therapy in CKD patients, depending on ferritin levels. When concomitant inflammation is present, ferritin and hepcidin are unlikely to predict the response to iron therapy since an increase in these parameters independent of iron status may induce hyporesponsiveness to iron supplementation [128]. Thus, measurement of CRP should be part of the routine hematological assessment of HD patients to allow the correct interpretation of data and therapeutic approach for IDA in CKD patients.

## 7. What Are Optimal Levels of Serum Ferritin for the Management of IDA in CKD Patients with Minor Inflammation?

Optimal levels of serum ferritin during iron therapy remain to be determined in CKD patients with and without apparent inflammation. Due to a lack of data in CKD patients with apparent inflammation, we herein discuss data reported in the literature for optimal serum ferritin levels to maintain tHb during iron therapy in CKD patients with IDA and minor inflammation. Table 4 summarizes data from studies reporting optimal serum ferritin levels in ND-CKD and dialysis patients with either minor inflammation [124,125,126] or low serum ferritin levels (<100 ng/mL) [105,164,165,166]. The patients in these studies were treated with either OIT or low dose of IIT and the majority of patients simultaneously received ESA. Based on the data, OIT or very low dose of IIT is as effective as the standard IIT to improve IDA in HD patients with minor inflammation or low serum ferritin levels. Optimal ferritin levels are both low in HD patients receiving OIT (30–115 ng/mL) and in those receiving IIT (<300 ng/mL), while they were lower in the OIT group than in the IIT group. In dialysis children, serum ferritin levels of 25–50 ng/mL were associated with an achievement of highest Hb levels, although mode of iron therapy and data for CRP are not available [105]. Our previous study showed that optimal serum ferritin levels were estimated to be 30–40 ng/mL in HD patients with normal CRP and that further increment of serum ferritin was associated with increased levels of serum hepcidin and decreased Hb levels [124]. As shown in Table 4, optimal serum ferritin levels are significantly lower in HD patients with minor inflammation receiving IIT than achieved serum ferritin targets in those with apparent inflammation receiving IIT (mean serum ferritin, 340–810 ng/mL) [114,120,123,136,167] but similar to those (mean, 238.5 ng/mL) in ND-CKD patients with weakly positive CRP (mean, 1.3 mg/dL) receiving IIT [118]. Thus, concomitant inflammation increases serum ferritin targets to maintain tHb, making IIT more preferable than OIT to maintain tHb in CKD patients. Of note is that optimal levels of serum ferritin in HD patients with minor inflammation receiving OIT or low dose of IIT (Table 4) are less than the cutoff serum ferritin levels of 160 ng/mL and 290 ng/mL to predict mild and severe iron overload in HD adults with positive CRP evaluated on magnetic resonance imaging (MRI) [168]. Taken together, these data suggest that optimal levels of serum ferritin during iron therapy for the management of IDA are quite low in CKD patients with minor inflammation as compared to those with apparent inflammation. Due to a small sample size, further studies are necessary to determine optimal levels of serum ferritin for the management of IDA in CKD patients with minor inflammation.

## 8. Role of Ferritin, Hepcidin and Inflammation for the Development of Complications in CKD

Ferritin deficiency is a risk factor for increased LVM index in ND-CKD patients [169]. In HD patients, high serum levels of ferritin were associated with progressive arterial stiffness, leading to atherosclerosis, in particular when serum ferritin was >500 ng/mL [170]. Higher levels of serum ferritin induced by aggressive IIT may be a cause of increased incidence of atherosclerosis in HD patients [171]. In dialysis children and adolescents with iron overload, ferritin levels showed a positive correlation with CRP, IL-6 and LVM [39]. On the other hand, decreased levels of serum hepcidin were associated with a higher LVM index in ND-CKD patients [172]. However, serum hepcidin levels were increased and positively correlated with brachial-ankle pulse wave velocity as the measurement of arterial stiffness in HD adults [173]. Similarly, carotid artery intima media thickness was positively correlated with serum hepcidin and CRP in diabetic HD patients, in whom hepcidin positively correlated with ferritin and inflammation markers (CRP, TNF-α and IL-6) [174]. Despite no relation between hepcidin, CRP, IL-6 and LVM [175], increased levels of serum hepcidin were associated with fatal and non-fatal cardiovascular events and inflammation was a significant confounder in the relation between hepcidin and all-cause mortality in HD patients [176]. These data suggest that both low and high levels of ferritin and hepcidin may be risk factors for the development of complications associated with CKD.

## 9. Safety Issues of Iron Therapy

### 9.1. Which is First Either OIT or IIT?

The French National Agency for the Safety of Medicines and Health Product (ANSM, Saint-Denis, France), Canadian guidelines and other investigators recommend that it is appropriate to use OIT in first intention due to its lower toxicity, regardless of ND-CKD or HD patients [52,126,177,178]. Several international guidelines also recommend that OIT should be administered first in ND-CKD patients [52,58,59], while other guidelines recommend that IIT be firstly used in HD patients [51,54,55,58,59]. Our previous study demonstrated that low dose of IIT could be a second line of iron supplementation for the management of IDA in HD patients with minor inflammation who failed to respond to OIT [124]. In support of our finding, IIT with ferrous saccharated (300–800 mg bolus once a month followed by 50 mg weekly for 3 months) was beneficial in 13 (76.4%) of the 17 HD patients with IDA who failed to maintain the tHb (10–11 g/dL) following OIT and ESA [179]. Total doses of IIT in our protocol [124] are lower than the low maintenance dose of IIT (iron gluconate 31.25 mg/week over 1 year) which could not prevent a risk of iron overload in HD patients [180] and far less than <250 mg of iron/month to avoid iron overload evaluated on MRI [181]. In our view, OIT should be first administered in CKD patients, in particular in those with minor inflammation, and be switched to IIT when patients fail to respond to OIT or severe adverse effects of OIT are present.

### 9.2. Adverse Effects of OIT and IIT

While oral iron is safer than IV iron, it is associated with reduced treatment adherence, due to gastrointestinal side effects [182,183]. The short-term studies reported no significant difference in prevalence of acute adverse effects between IIT and OIT in CKD patients [115,116]. However, IIT is more frequently associated with serious adverse events than OIT in ND-CKD patients [184]. IV iron, especially with high weekly doses, significantly increased a risk of infection and hospitalization and promoted oxidant stress, cardiovascular events and all-cause mortality in CKD patients [7,182,184,185,186]. Infection-related mortality was high in HD adults receiving IV iron dose of >1050 mg in 3 months or >2100 mg in 6 months [187]. Thus, the Kidney Disease Improving Global Outcomes (KDIGO) guidelines recommend not administering IIT during active infection [54]. Similarly, atherosclerotic change was correlated with serum levels of ferritin and doses of IV iron in HD patients [188]. OIT improved anemia in ND-CKD patients without affecting renal function [189], while IV iron sucrose increased proteinuria in ND-CKD patients, indicating that high doses of IV iron may aggravate kidney injury [190]. Repeated administration of IV iron in HD patients increased oxidative DNA injury and serum ferritin, suggesting that excess body iron stores caused by aggressive IIT might promote oxidative stress [191].

### 9.3. Iron Overload in CKD

Serum levels of ferritin are generally higher in HD patients receiving IIT compared to those receiving OIT [120,192]. In recent years, there is a trend for decreasing the use of ESA and increasing the use of IIT for CKD patients in the US [109]. Like CRP [15,62], serum ferritin levels and the doses of IV iron used were generally higher in HD patients of Western countries than in those of Japan [193,194]. Serum ferritin levels were increased in HD patients of Canada, Europe and the US from 1997 to 2011, compared to those of Japan [195]. Karaboyas et al. recently reported that during 2009–2015, median ferritin levels in HD adults were 718 ng/mL in the US, 405 ng/mL in Europe and 83 ng/mL in Japan [100]. In addition, mean serum ferritin levels of HD patients in the US in 2013 exceeded 800 ng/mL, with 18% of the patients exceeding ferritin of 1200 ng/mL [109]. Kalantar-Zadeh et al. defined iron overload as serum ferritin levels of >2000 ng/mL since serum ferritin may be increased up to 2000 ng/mL due to non-iron-related factors, including malnutrition-inflammation complex syndrome [40]. However, no clear evidence for iron overload was presented in this study.

Recently, several approaches using imaging for the quantification of liver iron concentration have been used to detect iron overload in CKD patients. Using superconducting quantum interference device for direct noninvasive magnetic measurements of non-heme hepatic iron content, 32.5% and 37.5% of HD patients had mild (liver iron content; LIC 400 to 1000 μg/g liver tissue) and severe iron overload (LIC > 1000 μg/g liver tissue), while 70% of these patients had serum ferritin levels of <500 ng/mL [196]. In this study, the best specificity/sensitivity ratio for serum ferritin to identify iron overload was proposed as >340 ng/mL [196]. Ghoti et al. showed that iron overload in the liver was detected using MRI in HD patients with serum ferritin levels of >1000 ng/mL receiving IIT [197]. However, Rostoker et al. using MRI showed that in HD adults with positive CRP, cutoff levels of serum ferritin were 160 ng/mL for mild (LIC > 50 μmol/g liver tissue) and 290 ng/mL for severe iron overload (LIC > 200 μmol/g liver tissue) [168]. Despite the presence of inflammation, these cutoff levels of serum ferritin to detect iron overload using MRI are significantly lower than upper limit of serum ferritin to avoid iron overload proposed by international guidelines (Table 2) [51,52,53,54,55,56,57,58,59,60]. The same research group using MRI recommended that the maximum amount of IV iron should be lowered to 250 mg/month to avoid iron overload [181]. In support of this recommendation, Bailie et al. reported that mortality was significantly higher in HD adults receiving IV iron dose of >300 mg/month than those receiving iron dose of <299 mg/month [186]. Thus, it remains to be determined whether international guidelines for the use of IV iron are safe enough to avoid a risk of iron overload [198].

To answer this question, accurate, noninvasive, rapid and inexpensive methods for evaluation of iron overload would be mandatory. In our view, international guidelines for upper limit of serum ferritin to avoid a risk of iron overload in CKD patients may need a revision according to the presence or absence of inflammation. Vaziri stated in his review [182] that compared with Western countries, the JSDT guidelines [53] for iron therapy in dialysis patients are far more conservative, while outcomes of the Japanese dialysis patients are as good as or better than those in American counterparts and that an approach with more conservative iron therapy should be considered. Efforts should be directed towards lowering serum ferritin targets to avoid the long-term adverse effects of iron overload-mediated toxicity especially in CKD patients with apparent inflammation.

### 9.4. Safer Treatment with Iron and Non-Iron Agents for the Management of IDA in CKD

There are a number of oral iron salts including ferrous sulphate, ferrous fumarate and ferrous gluconate, of which ferrous sulphate has been most frequently used in ND-CKD patients [183]. Recently, several oral iron agents have been used to reduce adverse effects for the management of IDA in CKD patients. Oral liposomal iron, a preparation of ferric pyrophosphate conveyed within a phospholipid membrane associated with ascorbic acid, is well absorbed from the gut and demonstrates high bioavailability with low side effects [118]. It is a safe and efficacious alternative to IV iron gluconate for the management of IDA in ND-CKD patients [118]. Oral heme iron polypeptide, a compound that uses the heme porphyrin ring to supply iron, can increase iron store and has similar efficacy to IV iron sucrose in ND-CKD [166] and HD patients [199]. As a new agent, low dose of oral ferric citrate, a compound comprising trivalent iron with citrate that functions as a phosphate binder, has been shown to improve IDA without inducing iron overload in hyperphosphatemic HD patients [200].

To avoid potential serious adverse effects of IIT, safer regimens with low dose of IV iron have been used for the management of IDA in CKD patients. Maintenance IV iron regimen in smaller doses at frequent intervals has been more efficacious and safer than large intermittent doses [201]. Continuous low dose of IV iron sucrose was more effective in maintaining tHb compared to the regimen with bolus high dose of IV iron in HD patients [202]. Low dose of iron sucrose (20 mg) at the end of every HD session was effective to maintain functional iron levels and ESA dose, thereby reducing iron overload in HD patients without serious adverse effects [203]. Weekly low dose (50 mg) of IV iron sucrose for 6 months maintained tHb and reduced the ESA dose without induction of high serum ferritin levels in HD patients [204]. In addition, low dose of IV ferric carboxymaltose appeared to be safe for the management of IDA in ND-CKD patients [98]. The efficacy and safety of IV ferumoxytol was comparable to IV iron sucrose in patients with varying degrees of renal function [205]. Intravenous low-molecular-weight iron dextran for 12 months was effective even in iron-pretreated HD patients [206]. Furthermore, ferric pyrophosphate citrate, a water-soluble iron salt administered via dialysate at a dose of 2 μmol/L (110 mg/L of Fe^3+^) to supply ∼5–7 mg of iron during the course of each dialysis session, was able to decrease the amount of IV iron needed for maintenance of tHb with a reduction of the ESA dose in HD patients [207].

As alternative treatment, non-iron agents that can inhibit hepcidin have recently been used for the management of IDA in CKD patients. Pentoxifylline, a methylxanthine derivative, inhibited phosphodiesterase, resulting in increased intracellular cyclic AMP, activation of protein kinase A and inhibition of IL and TNF synthesis as well as inflammation. Pentoxifylline reduced expression of IL-6 and ferritin and increased Hb and TSAT possibly through modulation of hepcidin in ND-CKD adults [208].

As other agents, an oral hypoxia-inducible factor (HIF)-prolyl hydroxylases (PHD) inhibitor—vadadustat (AKB-6548, Akebia Therapeutics Inc, Cambridge, MA, USA)—has been shown to increase Hb, mean absolute reticulocyte count and TIBC and reduced hepcidin and ferritin by stabilizing HIF in iron-replete ND-CKD patients receiving a minimum dose of OIT with or without ESA [209,210]. Roxadustat (FG-4592, FibroGen, San Francisco, CA, USA), other oral HIF-PHD inhibitor, decreased hepcidin, ferritin and TSAT and increased Hb and TIBC in iron-replete ND-CKD patients not receiving IIT and ESA [211]. Regardless of CRP and iron repletion, roxadustat increased Hb and decreased hepcidin in HD patients [212]. In this study, an increase in Hb was greater in HD patients receiving OIT or IIT than in those not receiving iron, while TSAT and CHr were decreased in the patients with no iron but unchanged in those receiving OIT or IIT. In addition, serum ferritin levels did not change in HD patients receiving OIT or IIT but decreased in those not receiving iron. Roxadustat can be used even in the presence of inflammation and leads to sufficiency of low-dose oral iron for anemia correction in CKD patients [212]. Other oral HIF-PHD inhibitor, daprodustat (GSK127886, GlaxoSmithKline, Philadelphia, PA, USA), decreased hepcidin, ferritin, serum iron and TSAT and increased Hb, total reticulocyte counts, TIBC and unsaturated iron-binding capacity in ND-CKD and dialysis patients [213]. In addition, HIF-PHD inhibitors can reduce iron dose to maintain tHb in HD patients and minimize iatrogenic iron overload from IV iron [212]. These non-iron agents can increase iron availability for effective erythropoiesis by decreasing ferritin and hepcidin and this effect is not affected by inflammation. Thus, they have a benefit for the management of IDA/FIDA even in CKD patients with apparent inflammation.

## 10. Minimizing a Risk of Inflammation as Therapeutic Strategy for IDA in CKD

Minimization of a risk of inflammation may reduce the required dose of iron therapy in HD patients [21]. Correctible causes of inflammation in CKD include tunneled dialysis catheters, AV grafts, catheter infection, periodontal disease, poor water quality and dialyzer incompatibility [62,214]. Ultrapure dialysate reduced serum levels of CRP and ferritin and improved iron utilization in HD patients [215]. Thus, OIT was as effective as IIT for the management of IDA in HD patients using ultrapure dialysate [165]. HD patients with catheters and AV grafts were associated with high inflammation markers and required a higher dose of ESA as compared to those with AV fistulae [21]. Obesity is frequently associated with IDA and inflammation in CKD patients [216,217]. Despite IDA, obesity increases the expression of hepcidin due to inflammation-induced hepcidin synthesis by adipose tissues [216]. Obese CKD patients frequently developed hyporesponsiveness to OIT due to increased hepcidin [217]. Therapeutic strategy for the management of IDA should include minimization of these risk factors of inflammation in CKD patients.

## 11. Conclusions

IDA induces resistance to ESA, poor QOL and increased mortality in CKD patients. Inflammation highly associated with CKD increases ferritin and hepcidin, which block iron absorption and efflux, leading to reduced iron availability for erythropoiesis and subsequent hyporesponsiveness to iron therapy and ESA. In the absence of inflammation, correlation between ferritin and hepcidin is robust to predict iron status and responsiveness to iron therapy. Diagnosis of IDA, criteria for initiation of iron therapy and upper limit of ferritin to predict iron overload are different among international guidelines for the management of IDA in CKD patients. Inflammation-mediated increase in ferritin and hepcidin independent of iron status affect these issues and reduces the predictive values of ferritin and hepcidin for the response to iron therapy. IIT has been considered to be superior to OIT for the management of IDA in CKD patients. While this may be true in the presence of apparent inflammation, OIT is as effective as IIT under minor inflammation. Many short-term studies show that aggressive IIT in CKD patients, especially in those with high inflammation, has been considered safe based on appearance of iron overload symptoms. Thus, iron overload can silently progress, leading to a risk of mortality in CKD patients. Currently, there is no evidence for how much iron is accumulated in the tissues and the long-term adverse effects of aggressive IIT including mortality in CKD patients. Upper limit of serum ferritin to predict iron overload using MRI is far less than that proposed by international guidelines for IDA in CKD patients. Accurate, non-invasive and rapid methods to detect iron overload other than MRI need to be established. Alteration of iron status such as IDA and iatrogenic iron overload and of expression of ferritin and hepcidin as well as inflammation may affect the development of complications and mortality in CKD patients. The management of IDA in CKD patients should differ, depending on the absence or presence of inflammation and include minimization of a risk of inflammation. Future well-powered studies using a large number of CKD patients with or without inflammation would be necessary to address the impact of inflammation on therapeutic strategies for the management of IDA in CKD patients.

## Figures and Tables

**Table 1:** Cutoff of hemoglobin (Hb) for diagnosis of anemia.

Age/Gender Groups	Hb Below (g/dL)
Children	
6 months to 4 years	<11.0
5 to 11 years	<11.5
12 to 14 years	<12.0
Adults	
Non-pregnant women ≥15 years	<12.0
Pregnant women ≥15 years	<11.0
Men ≥15 years	<13.0

**Table 2:** International clinical guidelines for diagnosis of IDA and upper limit of serum ferritin and TSAT in CKD patients.

Organization (Year)	Origin	ID/IDA	Recommended ID Cutoff Serum Ferritin (ng/mL)		TSAT (%)		Upper Limit of Serum Ferritin (ng/mL)	TSAT (%)	Reference
			ND	HD	ND	HD			
KDOQI (2007)	USA	ID/IDA	≤100	≤200	≤20	≤20	≤500	NA	[51]
CSN (2008)	Canada	ID/IDA	≤100	≤200	≤20	≤20	≤800	NA	[52]
JSDT (2008)	Japan	ID/IDA	≤100	≤100	≤20	≤20	≤800	≤50	[53]
		Children	≤100	≤100	≤20	≤20	NA	NA	
KDIGO (2012)	International	ID/IDA	≤500	≤500	≤30	≤30	≤500–800	NA	[54]
		Children	≤100	≤100	≤20	≤20	≤500–800	NA	
ERBP (2016)	Europe	ID/IDA	<100	<100	<20	<20	≤500	≤30	[55]
KHA-CARI (2013)	Australia	ID	<100	<100	<20	<20	≤500	NA	[56]
TPG (1996)	Taiwan	ID/IDA	≤300	≤300	≤30	≤30	≤800	≤50	[57]
NICE (2015)	UK	ID/IDA	≤100	≤100	≤20	≤20	<800	NA	[58,59]
UKRA (2017)	UK	ID/IDA	≤100	≤100	≤20	≤20	≤500–800	NA	[60]
		Children	≤100	≤100	≤20	≤20	≤500–800	NA	

CKD: chronic kidney disease; CSN: Canadian Society of Nephrology; ERBP: European Renal Best Practice; HD: hemodialysis; ID: iron deficiency; IDA: ID anemia; JSDT: The Japanese Society for Dialysis Therapy; KDIGO: The Kidney Disease, Improving Global Outcomes; KDOQI: The Kidney Disease Outcomes Quality Initiative; KHA-CARI: Kidney Health Australia-Caring for Australians with Renal Impairment; ND: non-dialysis, NICE: The National Institute for Health and Care Excellence; TPG: Taiwan Practice Guidelines; TSAT: transferrin saturation; UKRA: United Kingdom Renal Association. ID is defined as a decrease in the body iron stores. NA: not available.

**Table 3:** Impact of inflammation on the effect of intravenous and oral iron therapy for IDA in CKD patients.

Reference	Mode of Iron Therapy	Patients	Baseline Mean CRP (mg/dL)		Baseline Mean Ferritin (ng/mL)		ESA Use		Definition of Response to Iron Therapy	Response Rate or Maintained Hb Levels after Iron Therapy	
			OIT	IIT	OIT	IIT	OIT	IIT		OIT	IIT
Macdougall et al. [98,121]	OIT/IIT	ND-CKD	5.2	6.2; HFG, 6.7; LFG	57.3	56.4; HFG, 57.7; LFG	−	−	ΔHb ≥ 1 g/dL	32.1%	34.2% (LFG), 56.9% (HFG) *
Pisani et al. [118]	OIT/IIT	ND-CKD	1.2	1.3	71.4	67.7	+	+	ΔHb ≥ 0.6 g/dL	ΔHb 0.6 g/dL, 8.7%	ΔHb 1.0 g/dL, 33.7% **
Agarwal et al. [119]	OIT/IIT	ND-CKD	6.9	8.2	66.4	72.5	−	−	NA	ΔHb 0.2 g/dL	ΔHb 0.4 g/dL ***
Kalra et al. [120]	OIT/IIT	ND-CKD	8.6	9	98.8	95	−	−	NA	ΔHb 0.49 g/dL	ΔHb 0.94 g/dL *
Jenq et al. [122]	OIT/IIT	HD	4.8	12.4	181	348	+	+	ΔHt ≥ 3% from BL	12.5%	50% *
Stoves et al. [123]	OIT/IIT	ND-CKD	6	6	74	100	+	+	tHb 12 g/dL	Hb 12.2 (10.6–12.8) g/dL	Hb12.5 (11.6–13.3) g/dL
Takasawa et al. [124]	OIT	HD	0.11		29.5		+		ΔHb ≥ 2 g/dL	76.5%	
Ogawa et al. [125]	IIT	HD	0.06		50.6			+	tHb 10–11 g/dL	79.3%	
Sanai et al. [126]	OIT	HD	0.32		38		+		ΔHb ≥ 1 g/dL	100%	

BL: baseline, CKD: chronic kidney disease, CRP: C-reactive protein, ESA: erythropoiesis-stimulating agents, Hb: hemoglobin, HD: hemodialysis, Ht: hematocrit, IIT: intravenous iron therapy, ND: non-dialysis, OIT: oral iron therapy, tHb: target Hb. NA: not available. ΔHb and Ht are defined as the change in Hb and Ht before and after iron supplementation. HFG = high target ferritin group (400–600 ng/mL) receiving high dose of IIT (500–100 mg iron), LFG = low target ferritin group (100–200 ng/mL) receiving low dose of IIT (200 mg iron). * statistically significant vs. OIT. ** IIT produced a more rapid Hb increase than OIT. *** ΔHb did not differ between OIT and IIT groups but was only significant in IIT group.

**Table 4:** Optimal levels of serum ferritin during iron therapy in CKD patients with minor inflammation or lower serum ferritin levels.

Reference	Patients	Mode of Iron Therapy	Target Hb (g/dL)	ESA Dose Reduction after Iron Therapy	Mean Baseline CRP (mg/dL)	Mean Baseline Ferritin (ng/mL)	Optimal Serum Ferritin Levels after Iron Therapy (ng/mL)
Children							
van Stralen et al. [105]	PD/HD	57.3% received iron therapy ^†^	10.5–12.5	NA	NA	122	25–50
Adults							
Takasawa et al. [124]	HD	OIT	12–13	+	0.11	29.5	30–40
Ogawa et al. [125]	HD	Low dose IIT *	10–11	NA	0.06	50.6	<90
Sanai et al. [126]	HD	OIT	10–11	+	0.32	38	67.5 ± 44.0 ^#^
Lenga et al. [164]	HD	OIT	≥11	+	NA	72	≥100 (target)
Tsuchida et al. [165]	HD	OIT/IIT	10–11	+	NA	32.6; OIT	115.3 ± 28.1 ^#^; OIT
						57.8; IIT	183.5 ± 47.5 ^#^; IIT
Nagaraju et al. [166]	ND–CKD	OIT **/IIT	10.5–13	+	NA	71; OIT	85.5 (44–104) ^#^; OIT
						67; IIT	244 (71.5–298) ^#^; IIT

CKD: chronic kidney disease, CRP: C-reactive protein, ESA: erythropoiesis-stimulating agents, Hb: hemoglobin, HD: hemodialysis, IIT: intravenous iron therapy, ND: non-dialysis, OIT: oral iron therapy, PD: peritoneal dialysis. ^†^ Mode of iron therapy is unknown. * 40 mg of ferric saccharate/week for 2–6 weeks. ** Oral heme iron polypeptide. ^#^ Serum levels of ferritin achieved at the end of OIT or IIT and the data are expressed as mean ± SD. NA: not available.

## Abbreviations

AV	arteriovenous
BMP	bone morphogenetic protein
BMPR	BMP receptor
C/EBP	CCAAT enhancer-binding protein
CHr	content of hemoglobin in reticulocytes
CKD	chronic kidney disease
CP	ceruloplasmin
CREBH	cyclic-AMP-responsive-element-binding protein H
CRP	C-reactive protein
DCYTB	duodenal cytochrome b
DMT1	divalent metal transporter1
EPO	erythropoietin
ESA	erythropoiesis-stimulating agents
ESRD	end-stage renal disease
FIDA	functional IDA
FPN	ferroportin
FtH	heavy chain of ferritin
FtL	light chain of ferritin
GDF15	growth differentiation factor 15
GFR	glomerular filtration rate
Hb	hemoglobin
HD	hemodialysis
HIF	hypoxia-inducible factor
HO-1	heme oxygenase-1
HFE	hereditary hemochromatosis protein
HIF	hypoxia-inducible factor
Ht	hematocrit
IBD	inflammatory bowel disease
IDA	iron deficiency anemia
IIT	intravenous iron therapy
IL	interleukin
INF	interferon
IRE	iron responsive element
IRP	iron regulatory protein
JAK	Janus kinase
LN	lupus nephritis
LPS	lipopolysaccharide
LVM	left ventricular mass
mHJV	membrane-anchored hemojuvelin
MRI	magnetic resonance imaging
MT-2	matriptase-2
ND	non-dialysis
NF	nuclear factor
NO	nitric oxide
OIT	oral iron therapy
%Hypo	percentage of hypochromic red cells
PD	peritoneal dialysis
PHD	prolyl hydroxylase
PGC-1α	peroxisome proliferator-activated receptor γ coactivator-1α
QOL	quality of life
RA	rheumatoid arthritis
sHJV	soluble HJV
SLE	systemic lupus erythematosus
SMAD	small mothers against decapentaplegic proteins
STAT	signal transducers and activators of transcription
sTfR	soluble transferrin receptor
Tf	transferrin
tHb	target Hb
TIBC	total iron binding capacity
TNF	tumor necrosis factor
TSAT	Tf saturation

## References

[1] Lopez A., Cacoub P., Macdougall I.C., Peyrin-Biroulet L. (2016). Iron deficiency anaemia. Lancet.

[2] Levi M., Rosselli M., Simonetti M., Brignoli O., Cancian M., Masotti A., Pegoraro V., Cataldo N., Heiman F., Chelo M. (2016). Epidemiology of iron deficiency anaemia in four European countries: A population-based study in primary care. Eur. J. Haematol..

[3] Atkinson M.A., Furth S.L. (2011). Anemia in children with chronic kidney disease. Nat. Rev. Nephrol..

[4] Iimori S., Naito S., Noda Y., Nishida H., Kihira H., Yui N., Okado T., Sasaki S., Uchida S., Rai T. (2015). Anaemia management and mortality risk in newly visiting patients with chronic kidney disease in Japan: The CKD-ROUTE study. Nephrology (Carlton).

[5] Geissler C., Singh M. (2011). Iron, meat and health. Nutrients.

[6] Torti F.M., Torti S.V. (2002). Regulation of ferritin genes and protein. Blood.

[7] Kuragano T., Matsumura O., Matsuda A., Hara T., Kiyomoto H., Murata T., Kitamura K., Fujimoto S., Hase H., Joki N. (2014). Association between hemoglobin variability, serum ferritin levels, and adverse events/mortality in maintenance hemodialysis patients. Kidney Int..

[8] Ogawa C., Tsuchiya K., Kanda F., Maeda T. (2014). Low levels of serum ferritin lead to adequate hemoglobin levels and good survival in hemodialysis patients. Am. J. Nephrol..

[9] Zitt E., Sturm G., Kronenberg F., Neyer U., Knoll F., Lhotta K., Weiss G. (2014). Iron supplementation and mortality in incident dialysis patients: An observational study. PLoS ONE.

[10] Hentze M.W., Muckenthaler M.U., Galy B., Camaschella C. (2010). Two to tango: Regulation of mammalian iron metabolism. Cell.

[11] Ganz T. (2013). Systemic iron homeostasis. Physiol. Rev..

[12] Ueda N., Takasawa K. (2017). Role of hepcidin-25 in chronic kidney disease: Anemia and beyond. Curr. Med. Chem..

[13] Antonelli M., Kushner I. (2017). It’s time to redefine inflammation. FASEB J..

[14] Suchdev P.S., Williams A.M., Mei Z., Flores-Ayala R., Pasricha S.R., Rogers L.M., Namaste S.M. (2017). Assessment of iron status in settings of inflammation: Challenges and potential approaches. Am. J. Clin. Nutr..

[15] Kawaguchi T., Tong L., Robinson B.M., Sen A., Fukuhara S., Kurokawa K., Canaud B., Lameire N., Port F.K., Pisoni R.L. (2011). C-reactive protein and mortality in hemodialysis patients: The Dialysis Outcomes and Practice Patterns Study (DOPPS). Nephron Clin. Pract..

[16] Jairam A., Das R., Aggarwal P.K., Kohli H.S., Gupta K.L., Sakhuja V., Jha V. (2010). Iron status, inflammation and hepcidin in ESRD patients: The confounding role of intravenous iron therapy. Indian J. Nephrol..

[17] Małyszko J., Koc-Żórawska E., Levin-Iaina N., Małyszko J., Koźmiński P., Kobus G., Myśliwiec M. (2012). New parameters in iron metabolism and functional iron deficiency in patients on maintenance hemodialysis. Pol. Arch. Med. Wewn..

[18] Łukaszyk E., Łukaszyk M., Koc-Żórawska E., Tobolczyk J., Bodzenta-Łukaszyk A., Małyszko J. (2015). Iron status and inflammation in early stages of chronic kidney disease. Kidney Blood Press. Res..

[19] Gangat N., Wolanskyj A.P. (2013). Anemia of chronic disease. Semin. Hematol..

[20] Susantitaphong P., Alqahtani F., Jaber B.L. (2014). Efficacy and safety of intravenous iron therapy for functional iron deficiency anemia in hemodialysis patients: A meta-analysis. Am. J. Nephrol..

[21] El-Khatib M., Duncan H.J., Kant K.S. (2006). Role of C-reactive protein, reticulocyte haemoglobin content and inflammatory markers in iron and erythropoietin administration in dialysis patients. Nephrology (Carlton).

[22] Singh A.K., Coyne D.W., Shapiro W., Rizkala A.R., DRIVE Study Group (2007). Predictors of the response to treatment in anemic hemodialysis patients with high serum ferritin and low transferrin saturation. Kidney Int..

[23] Malyszko J., Malyszko J.S., Mysliwiec M. (2009). Hyporesponsiveness to erythropoietin therapy in hemodialyzed patients: Potential role of prohepcidin, hepcidin, and inflammation. Ren. Fail..

[24] Naz N., Moriconi F., Ahmad S., Amanzada A., Khan S., Mihm S., Ramadori G., Malik I.A. (2013). Ferritin L is the sole serum ferritin constituent and a positive hepatic acute-phase protein. Shock.

[25] Pham C.G., Bubici C., Zazzeroni F., Papa S., Jones J., Alvarez K., Jayawardena S., De Smaele E., Cong R., Beaumont C. (2004). Ferritin heavy chain upregulation by NF-kB inhibits TNFα-induced apoptosis by suppressing reactive oxygen species. Cell.

[26] Recalcati S., Taramelli D., Conte D., Cairo G. (1998). Nitric oxide-mediated induction of ferritin synthesis in J774 macrophages by inflammatory cytokines: Role of selective iron regulatory protein-2 downregulation. Blood.

[27] Nakanishi T., Kuragano T., Nanami M., Otaki Y., Nonoguchi H., Hasuike Y. (2010). Importance of ferritin for optimizing anemia therapy in chronic kidney disease. Am. J. Nephrol..

[28] Zhao N., Zhang A.S., Enns C.A. (2013). Iron regulation by hepcidin. J. Clin. Investig..

[29] Zhao N., Maxson J.E., Zhang R.H., Wahedi M., Enns C.A., Zhang A.S. (2016). Neogenin facilitates the induction of hepcidin expression by hemojuvelin in the liver. J. Biol. Chem..

[30] Zhao N., Nizzi C.P., Anderson S.A., Wang J., Ueno A., Tsukamoto H., Eisenstein R.S., Enns C.A., Zhang A.S. (2015). Low intracellular iron increases the stability of matriptase-2. J. Biol. Chem..

[31] Silvestri L., Pagani A., Camaschella C. (2008). Furin-mediated release of soluble hemojuvelin: A new link between hypoxia and iron homeostasis. Blood.

[32] Wichaiyo S., Yatmark P., Morales Vargas R.E., Sanvarinda P., Svasti S., Fucharoen S., Morales N.P. (2015). Effect of iron overload on furin expression in wild-type and β-thalassemic mice. Toxicol. Rep..

[33] Canali S., Vecchi C., Garuti C., Montosi G., Babitt J.L., Pietrangelo A. (2016). The SMAD pathway is required for hepcidin response during endoplasmic reticulum stress. Endocrinology.

[34] Shin D.Y., Chung J., Joe Y., Pae H.O., Chang K.C., Cho G.J., Ryter S.W., Chung H.T. (2012). Pretreatment with CO-releasing molecules suppresses hepcidin expression during inflammation and endoplasmic reticulum stress through inhibition of the STAT3 and CREBH pathways. Blood.

[35] Vecchi C., Montosi G., Zhang K., Lamberti I., Duncan S.A., Kaufman R.J., Pietrangelo A. (2009). ER stress controls iron metabolism through induction of hepcidin. Science.

[36] Meynard D., Sun C.C., Wu Q., Chen W., Chen S., Nelson C.N., Waters M.J., Babitt J.L., Lin H.Y. (2013). Inflammation regulates TMPRSS6 expression via STAT5. PLoS ONE.

[37] Qian J., Chen S., Huang Y., Shi X., Liu C. (2013). PGC-1α regulates hepatic hepcidin expression and iron homeostasis in response to inflammation. Mol. Endocrinol..

[38] Kanamori Y., Murakami M., Sugiyama M., Hashimoto O., Matsui T., Funaba M. (2017). Interleukin-1β (IL-1β) transcriptionally activates hepcidin by inducing CCAAT enhancer-binding protein δ (C/EBPδ) expression in hepatocytes. J. Biol. Chem..

[39] Ruiz-Jaramillo Mde L., Guízar-Mendoza J.M., Amador-Licona N., Gutiérrez-Navarro Mde J., Hernández-González M.A., Dubey-Ortega L.A., Solorio-Meza S.E. (2011). Iron overload as cardiovascular risk factor in children and adolescents with renal disease. Nephrol. Dial. Transplant..

[40] Kalantar-Zadeh K., Rodriguez R.A., Humphreys M.H. (2004). Association between serum ferritin and measures of inflammation, nutrition and iron in haemodialysis patients. Nephrol. Dial. Transplant..

[41] Hayes W. (2018). Measurement of iron status in chronic kidney disease. Pediatr. Nephrol..

[42] Kemna E., Pickkers P., Nemeth E., van der Hoeven H., Swinkels D. (2005). Time-course analysis of hepcidin, serum iron, and plasma cytokine levels in humans injected with LPS. Blood.

[43] Formanowicz D., Formanowicz P. (2012). Transferrin changes in haemodialysed patients. Int. Urol. Nephrol..

[44] Gaweda A.E. (2017). Markers of iron status in chronic kidney disease. Hemodial. Int..

[45] Hackeng C.M., Beerenhout C.M., Hermans M., Van der Kuy P.H., Van der Dussen H., Van Dieijen-Visser M.P., Hamulyák K., Van der Sande F.M., Leunissen K.M., Kooman J.P. (2004). The relationship between reticulocyte hemoglobin content with C-reactive protein and conventional iron parameters in dialysis patients. J. Nephrol..

[46] Bovy C., Tsobo C., Crapanzano L., Rorive G., Beguin Y., Albert A., Paulus J.M. (1999). Factors determining the percentage of hypochromic red blood cells in hemodialysis patients. Kidney Int..

[47] Zaritsky J., Young B., Wang H.J., Westerman M., Olbina G., Nemeth E., Ganz T., Rivera S., Nissenson A.R., Salusky I.B. (2009). Hepcidin—A potential novel biomarker for iron status in chronic kidney disease. Clin. J. Am. Soc. Nephrol..

[48] Mercadal L., Metzger M., Haymann J.P., Thervet E., Boffa J.J., Flamant M., Vrtovsnik F., Houillier P., Froissart M., Stengel B. (2014). The relation of hepcidin to iron disorders, inflammation and hemoglobin in chronic kidney disease. PLoS ONE.

[49] Zaritsky J., Young B., Gales B., Wang H.J., Rastogi A., Westerman M., Nemeth E., Ganz T., Salusky I.B. (2010). Reduction of serum hepcidin by hemodialysis in pediatric and adult patients. Clin. J. Am. Soc. Nephrol..

[50] Ford B.A., Eby C.S., Scott M.G., Coyne D.W. (2010). Intra-individual variability in serum hepcidin precludes its use as a marker of iron status in hemodialysis patients. Kidney Int..

[51] National Kidney Foundation (2006). KDOQI Clinical practice guidelines and clinical practice recommendations for anemia in chronic kidney disease. Am. J. Kidney Dis..

[52] Madore F., White C.T., Foley R.N., Barrett B.J., Moist L.M., Klarenbach S.W., Culleton B.F., Tonelli M., Manns B.J., Canadian Society of Nephrology (2008). Clinical practice guidelines for assessment and management of iron deficiency. Kidney Int. Suppl..

[53] Tsubakihara Y., Nishi S., Akiba T., Hirakata H., Iseki K., Kubota M., Kuriyama S., Komatsu Y., Suzuki M., Nakai S. (2010). 2008 Japanese Society for Dialysis Therapy: Guidelines for renal anemia in chronic kidney disease. Ther. Apher. Dial..

[54] The Kidney Disease: Improving Global Outcomes (KDIGO) Anemia Work Group (2012). KDIGO clinical practice guideline for anemia in chronic kidney disease. Kidney Int. Suppl..

[55] Locatelli F., Bárány P., Covic A., De Francisco A., Del Vecchio L., Goldsmith D., Hörl W., London G., Vanholder R., Van Biesen W. (2013). Kidney disease: Improving global outcomes guidelines on anaemia management in chronic kidney disease: A European Renal Best Practice position statement. Nephrol. Dial. Transplant..

[56] Macginley R., Walker R., Irving M. (2013). KHA-CARI Guideline: Use of iron in chronic kidney disease patients. Nephrology (Carlton).

[57] Hung S.C., Kuo K.L., Tarng D.C., Hsu C.C., Wu M.S., Huang T.P. (2014). Anaemia management in patients with chronic kidney disease: Taiwan practice guidelines. Nephrology (Carlton).

[58] National Clinical Guideline Centre (UK) (2015). Anaemia Management in Chronic Kidney Disease: Partial Update 2015. National Institute for Health and Care Excellence (NICE): Clinical Guideline.

[59] Ratcliffe L.E., Thomas W., Glen J., Padhi S., Pordes B.A., Wonderling D., Connell R., Stephens S., Mikhail A.I., Fogarty D.G. (2016). Diagnosis and management of iron deficiency in CKD: A summary of the NICE Guideline Recommendations and Their Rationale. Am. J. Kidney Dis..

[60] Mikhail A., Brown C., Williams J.A., Mathrani V., Shrivastava R., Evans J., Isaac H., Bhandari S. (2017). Renal association clinical practice guideline on anaemia of chronic kidney disease. BMC Nephrol..

[61] Hamano T., Fujii N., Hayashi T., Yamamoto H., Iseki K., Tsubakihara Y. (2015). Thresholds of iron markers for iron deficiency erythropoiesis-finding of the Japanese nationwide dialysis registry. Kidney Int. Suppl..

[62] Bazeley J., Bieber B., Li Y., Morgenstern H., de Sequera P., Combe C., Yamamoto H., Gallagher M., Port F.K., Robinson B.M. (2011). C-reactive protein and prediction of 1-year mortality in prevalent hemodialysis patients. Clin. J. Am. Soc. Nephrol..

[63] Ethier J., Mendelssohn D.C., Elder S.J., Hasegawa T., Akizawa T., Akiba T., Canaud B.J., Pisoni R.L. (2008). Vascular access use and outcomes: An international perspective from the Dialysis Outcomes and Practice Patterns Study. Nephrol. Dial. Transplant..

[64] Pisoni R.L., Arrington C.J., Albert J.M., Ethier J., Kimata N., Krishnan M., Rayner H.C., Saito A., Sands J.J., Saran R. (2009). Facility hemodialysis vascular access use and mortality in countries participating in DOPPS: An instrumental variable analysis. Am. J. Kidney Dis..

[65] Banerjee T., Kim S.J., Astor B., Shafi T., Coresh J., Powe N.R. (2014). Vascular access type, inflammatory markers, and mortality in incident hemodialysis patients: The Choices for Healthy Outcomes in Caring for End-Stage Renal Disease (CHOICE) Study. Am. J. Kidney Dis..

[66] Baracco R., Saadeh S., Valentini R., Kapur G., Jain A., Mattoo T.K. (2011). Iron deficiency in children with early chronic kidney disease. Pediatr. Nephrol..

[67] Cusick S.E., Mei Z., Freedman D.S., Looker A.C., Ogden C.L., Gunter E., Cogswell M.E. (2008). Unexplained decline in the prevalence of anemia among US children and women between 1988–1994 and 1999–2002. Am. J. Clin. Nutr..

[68] Akbari M., Moosazadeh M., Tabrizi R., Khatibi S.R., Khodadost M., Heydari S.T., Tahami A.N., Lankarani K.B. (2017). Estimation of iron deficiency anemia in Iranian children and adolescents: A systematic review and meta-analysis. Hematology.

[69] Fishbane S., Pollack S., Feldman H.I., Joffe M.M. (2009). Iron indices in chronic kidney disease in the National Health and Nutritional Examination Survey 1988–2004. Clin. J. Am. Soc. Nephrol..

[70] Cappellini M.D., Comin-Colet J., de Francisco A., Dignass A., Doehner W., Lam C.S., Macdougall I.C., Rogler G., Camaschella C., Kadir R. (2017). Iron deficiency across chronic inflammatory conditions: International expert opinion on definition, diagnosis, and management. Am. J. Hematol..

[71] Centers for Disease Control and Prevention (CDC) (2002). Iron deficiency—United States, 1999–2000. MMWR Morb. Mortal. Wkly. Rep..

[72] Haupt L., Weyers R. (2016). Determination of functional iron deficiency status in haemodialysis patients in central South Africa. Int. J. Lab. Hematol..

[73] Knight T.G., Ryan K., Schaefer C.P., D’Sylva L., Durden E.D. (2010). Clinical and economic outcomes in Medicare beneficiaries with stage 3 or stage 4 chronic kidney disease and anemia: The role of intravenous iron therapy. J. Manag. Care Pharm..

[74] Kovesdy C.P., Estrada W., Ahmadzadeh S., Kalantar-Zadeh K. (2009). Association of markers of iron stores with outcomes in patients with nondialysis-dependent chronic kidney disease. Clin. J. Am. Soc. Nephrol..

[75] Bross R., Zitterkoph J., Pithia J., Benner D., Rambod M., Kovesdy C., Kopple J.D., Kalantar-Zadeh K. (2009). Association of serum total iron-binding capacity and its changes over time with nutritional and clinical outcomes in hemodialysis patients. Am. J. Nephrol..

[76] Cuevas X., García F., Martín-Malo A., Fort J., Lladós F., Lozano J., Pérez-García R. (2012). Risk factors associated with cardiovascular morbidity and mortality in Spanish incident hemodialysis patients: Two-year results from the ANSWER study. Blood Purif..

[77] Kleine C.E., Soohoo M., Ranasinghe O.N., Park C., Marroquin M.V., Obi Y., Rhee C.M., Moradi H., Kovesdy C.P., Kalantar-Zadeh K. (2018). Association of pre-end-stage renal disease hemoglobin with early dialysis outcomes. Am. J. Nephrol..

[78] Roberts T.L., Foley R.N., Weinhandl E.D., Gilbertson D.T., Collins A.J. (2006). Anaemia and mortality in haemodialysis patients: Interaction of propensity score for predicted anaemia and actual haemoglobin levels. Nephrol. Dial. Transplant..

[79] Fort J., Cuevas X., García F., Pérez-García R., Lladós F., Lozano J., Martín-Malo A., ANSWER Study (2010). Mortality in incident haemodialysis patients: Time-dependent haemoglobin levels and erythropoiesis-stimulating agent dose are independent predictive factors in the ANSWER study. Nephrol. Dial. Transplant..

[80] Avram M.M., Blaustein D., Fein P.A., Goel N., Chattopadhyay J., Mittman N. (2003). Hemoglobin predicts long-term survival in dialysis patients: A 15-year single-center longitudinal study and a correlation trend between prealbumin and hemoglobin. Kidney Int. Suppl..

[81] Macdougall I.C., Tomson C.R., Steenkamp M., Ansell D. (2010). Relative risk of death in UK haemodialysis patients in relation to achieved haemoglobin from 1999 to 2005: An observational study using UK Renal Registry data incorporating 30,040 patient-years of follow-up. Nephrol. Dial. Transplant..

[82] Shi Z., Zhen S., Zhou Y., Taylor A.W. (2017). Hb level, iron intake and mortality in Chinese adults: A 10-year follow-up study. Br. J. Nutr..

[83] Rheault M.N., Molony J.T., Nevins T., Herzog C.A., Chavers B.M. (2017). Hemoglobin of 12 g/dl and above is not associated with increased cardiovascular morbidity in children on hemodialysis. Kidney Int..

[84] Ishani A., Guo H., Gilbertson D.T., Liu J., Dunning S., Collins A.J., Foley R.N. (2007). Time to target haemoglobin concentration (11 g/dl)—Risk of hospitalization and mortality among incident dialysis patients. Nephrol. Dial. Transplant..

[85] Shoji T., Niihata K., Fukuma S., Fukuhara S., Akizawa T., Inaba M. (2017). Both low and high serum ferritin levels predict mortality risk in hemodialysis patients without inflammation. Clin. Exp. Nephrol..

[86] Kalantar-Zadeh K., McAllister C.J., Lehn R.S., Liu E., Kopple J.D. (2004). A low serum iron level is a predictor of poor outcome in hemodialysis patients. Am. J. Kidney Dis..

[87] Koo H.M., Kim C.H., Doh F.M., Lee M.J., Kim E.J., Han J.H., Han J.S., Oh H.J., Park J.T., Han S.H. (2014). The relationship of initial transferrin saturation to cardiovascular parameters and outcomes in patients initiating dialysis. PLoS ONE.

[88] Kalantar-Zadeh K., Lee G.H., Miller J.E., Streja E., Jing J., Robertson J.A., Kovesdy C.P. (2009). Predictors of hyporesponsiveness to erythropoiesis-stimulating agents in hemodialysis patients. Am. J. Kidney Dis..

[89] Ishigami J., Onishi T., Shikuma S., Akita W., Mori Y., Asai T., Kuwahara M., Sasaki S., Tsukamoto Y. (2013). The impact of hyporesponsiveness to erythropoietin-stimulating agents on time-dependent mortality risk among CKD stage 5D patients: A single-center cohort study. Clin. Exp. Nephrol..

[90] Liu S., Zhang D.L., Guo W., Cui W.Y., Liu W.H. (2012). Left ventricular mass index and aortic arch calcification score are independent mortality predictors of maintenance hemodialysis patients. Hemodial. Int..

[91] Ishida J.H., Johansen K.L. (2014). Iron and infection in hemodialysis patients. Semin. Dial..

[92] Jenq C.C., Hsu C.W., Huang W.H., Chen K.H., Lin J.L., Lin-Tan D.T. (2009). Serum ferritin levels predict all-cause and infection-cause 1-year mortality in diabetic patients on maintenance hemodialysis. Am. J. Med. Sci..

[93] Kessler M., Hoen B., Mayeux D., Hestin D., Fontenaille C. (1993). Bacteremia in patients on chronic hemodialysis. A multicenter prospective survey. Nephron.

[94] Park K.S., Ryu G.W., Jhee J.H., Kim H.W., Park S., Lee S.A., Kwon Y.E., Kim Y.L., Ryu H.J., Lee M.J. (2015). Serum ferritin predicts mortality regardless of inflammatory and nutritional status in patients starting dialysis: A prospective cohort study. Blood Purif..

[95] Kim T., Streja E., Soohoo M., Rhee C.M., Eriguchi R., Kim T.W., Chang T.I., Obi Y., Kovesdy C.P., Kalantar-Zadeh K. (2017). Serum ferritin variations and mortality in incident hemodialysis patients. Am. J. Nephrol..

[96] Brookhart M.A., Freburger J.K., Ellis A.R., Winkelmayer W.C., Wang L., Kshirsagar A.V. (2016). Comparative short-term safety of sodium ferric gluconate versus iron sucrose in hemodialysis patients. Am. J. Kidney Dis..

[97] Hoen B., Paul-Dauphin A., Kessler M. (2002). Intravenous iron administration does not significantly increase the risk of bacteremia in chronic hemodialysis patients. Clin. Nephrol..

[98] Macdougall I.C., Bock A.H., Carrera F., Eckardt K.U., Gaillard C., Van Wyck D., Roubert B., Nolen J.G., Roger S.D., FIND-CKD Study Investigators (2014). FIND-CKD: A randomized trial of intravenous ferric carboxymaltose versus oral iron in patients with chronic kidney disease and iron deficiency anaemia. Nephrol. Dial. Transplant..

[99] Kalantar-Zadeh K., Regidor D.L., McAllister C.J., Michael B., Warnock D.G. (2005). Time-dependent associations between iron and mortality in hemodialysis patients. J. Am. Soc. Nephrol..

[100] Karaboyas A., Morgenstern H., Pisoni R.L., Zee J., Vanholder R., Jacobson S.H., Inaba M., Loram L.C., Port F.K., Robinson B.M. (2018). Association between serum ferritin and mortality: Findings from the USA, Japan and European Dialysis Outcomes and Practice Patterns Study. Nephrol. Dial. Transplant..

[101] Floege J., Gillespie I.A., Kronenberg F., Anker S.D., Gioni I., Richards S., Pisoni R.L., Robinson B.M., Marcelli D., Froissart M. (2015). Development and validation of a predictive mortality risk score from a European hemodialysis cohort. Kidney Int..

[102] Chauveau P., Level C., Lasseur C., Bonarek H., Peuchant E., Montaudon D., Vendrely B., Combe C. (2003). C-reactive protein and procalcitonin as markers of mortality in hemodialysis patients: A 2-year prospective study. J. Ren. Nutr..

[103] Leonard M.B., Donaldson L.A., Ho M., Geary D.F. (2003). A prospective cohort study of incident maintenance dialysis in children: An NAPRTC study. Kidney Int..

[104] Chavers B.M., Roberts T.L., Herzog C.A., Collins A.J., St Peter W.L. (2004). Prevalence of anemia in erythropoietin-treated pediatric as compared to adult chronic dialysis patients. Kidney Int..

[105] Van Stralen K.J., Krischock L., Schaefer F., Verrina E., Groothoff J.W., Evans J., Heaf J., Ivanov D., Kostic M., Maringhini S. (2012). Prevalence and predictors of the sub-target Hb level in children on dialysis. Nephrol. Dial. Transplant..

[106] Dmitrieva O., de Lusignan S., Macdougall I.C., Gallagher H., Tomson C., Harris K., Desombre T., Goldsmith D. (2013). Association of anaemia in primary care patients with chronic kidney disease: Cross sectional study of quality improvement in chronic kidney disease (QICKD) trial data. BMC Nephrol..

[107] Freburger J.K., Ng L.J., Bradbury B.D., Kshirsagar A.V., Brookhart M.A. (2012). Changing patterns of anemia management in US hemodialysis patients. Am. J. Med..

[108] Miskulin D.C., Zhou J., Tangri N., Bandeen-Roche K., Cook C., Ephraim P.L., Crews D.C., Scialla J.J., Sozio S.M., Shafi T. (2013). Trends in anemia management in US hemodialysis patients 2004–2010. BMC Nephrol..

[109] Charytan D.M., Pai A.B., Chan C.T., Coyne D.W., Hung A.M., Kovesdy C.P., Fishbane S., Dialysis Advisory Group of the American Society of Nephrology (2015). Considerations and challenges in defining optimal iron utilization in hemodialysis. J. Am. Soc. Nephrol..

[110] Hassan R.H., Kandil S.M., Zeid M.S., Zaki M.E., Fouda A.E. (2017). Kidney injury in infants and children with iron-deficiency anemia before and after iron treatment. Hematology.

[111] Streja E., Kovesdy C.P., Greenland S., Kopple J.D., McAllister C.J., Nissenson A.R., Kalantar-Zadeh K. (2008). Erythropoietin, iron depletion, and relative thrombocytosis: A possible explanation for hemoglobin-survival paradox in hemodialysis. Am. J. Kidney Dis..

[112] Peyrin-Biroulet L., Williet N., Cacoub P. (2015). Guidelines on the diagnosis and treatment of iron deficiency across indications: A systematic review. Am. J. Clin. Nutr..

[113] Takasawa K., Takaeda C., Maeda T., Ueda N. (2015). Hepcidin-25, mean corpuscular volume, and ferritin as predictors of response to oral iron supplementation in hemodialysis patients. Nutrients.

[114] Peters N.O., Jay N., Cridlig J., Rostoker G., Frimat L. (2017). Targets for adapting intravenous iron dose in hemodialysis: A proof of concept study. BMC Nephrol..

[115] Rozen-Zvi B., Gafter-Gvili A., Paul M., Leibovici L., Shpilberg O., Gafter U. (2008). Intravenous versus oral iron supplementation for the treatment of anemia in CKD: Systematic review and meta-analysis. Am. J. Kidney Dis..

[116] Albaramki J., Hodson E.M., Craig J.C., Webster A.C. (2012). Parenteral versus oral iron therapy for adults and children with chronic kidney disease. Cochrane Database Syst. Rev..

[117] Qunibi W.Y., Martinez C., Smith M., Benjamin J., Mangione A., Roger S.D. (2011). A randomized controlled trial comparing intravenous ferric carboxymaltose with oral iron for treatment of iron deficiency anaemia of non-dialysis-dependent chronic kidney disease patients. Nephrol. Dial. Transplant..

[118] Pisani A., Riccio E., Sabbatini M., Andreucci M., Del Rio A., Visciano B. (2015). Effect of oral liposomal iron versus intravenous iron for treatment of iron deficiency anaemia in CKD patients: A randomized trial. Nephrol. Dial. Transplant..

[119] Agarwal R., Rizkala A.R., Bastani B., Kaskas M.O., Leehey D.J., Besarab A. (2006). A randomized controlled trial of oral versus intravenous iron in chronic kidney disease. Am. J. Nephrol..

[120] Kalra P.A., Bhandari S., Saxena S., Agarwal D., Wirtz G., Kletzmayr J., Thomsen L.L., Coyne D.W. (2016). A randomized trial of iron isomaltoside 1000 versus oral iron in non-dialysis-dependent chronic kidney disease patients with anaemia. Nephrol. Dial. Transplant..

[121] Macdougall I.C., Bock A.H., Carrera F., Eckardt K.U., Gaillard C., Wyck D.V., Meier Y., Larroque S., Perrin A., Roger S.D. (2017). Erythropoietic response to oral iron in patients with nondialysis-dependent chronic kidney disease in the FIND-CKD trial. Clin. Nephrol..

[122] Jenq C.C., Tian Y.C., Wu H.H., Hsu P.Y., Huang J.Y., Chen Y.C., Fang J.T., Yang C.W. (2008). Effectiveness of oral and intravenous iron therapy in haemodialysis patients. Int. J. Clin. Pract..

[123] Stoves J., Inglis H., Newstead C.G. (2001). A randomized study of oral vs intravenous iron supplementation in patients with progressive renal insufficiency treated with erythropoietin. Nephrol. Dial. Transplant..

[124] Takasawa K., Takaeda C., Wada T., Ueda N. (2018). Optimal serum ferritin levels for iron deficiency anemia during oral iron therapy (OIT) in Japanese hemodialysis patients with minor inflammation and benefit of intravenous iron therapy for OIT-nonresponders. Nutrients.

[125] Ogawa C., Tsuchiya K., Tomosugi N., Kanda F., Maeda K., Maeda T. (2017). Low levels of serum ferritin and moderate transferrin saturation lead to adequate hemoglobin levels in hemodialysis patients, retrospective observational study. PLoS ONE.

[126] Sanai T., Ono T., Fukumitsu T. (2017). Beneficial effects of oral iron in Japanese patients on hemodialysis. Intern. Med..

[127] Kooistra M.P., Niemantsverdriet E.C., van Es A., Mol-Beermann N.M., Struyvenberg A., Marx J.J. (1998). Iron absorption in erythropoietin-treated haemodialysis patients: Effects of iron availability, inflammation and aluminium. Nephrol. Dial. Transplant..

[128] Nakanishi T., Kuragano T., Kaibe S., Nagasawa Y., Hasuike Y. (2012). Should we reconsider iron administration based on prevailing ferritin and hepcidin concentrations?. Clin. Exp. Nephrol..

[129] Owen W.F., Lowrie E.G. (1998). C-reactive protein as an outcome predictor for maintenance hemodialysis patients. Kidney Int..

[130] Kalender B., Mutlu B., Ersöz M., Kalkan A., Yilmaz A. (2002). The effects of acute phase proteins on serum albumin, transferrin and haemoglobin in haemodialysis patients. Int. J. Clin. Pract..

[131] Heidari B., Fazli M.R., Misaeid M.A., Heidari P., Hakimi N., Zeraati A.A. (2015). A linear relationship between serum high-sensitive C-reactive protein and hemoglobin in hemodialysis patients. Clin. Exp. Nephrol..

[132] Chan K.E., Lafayette R.A., Whittemore A.S., Hlatky M.A., Moran J. (2008). Facility factors dominate the ability to achieve target haemoglobin levels in haemodialysis patients. Nephrol. Dial. Transplant..

[133] Sharain K., Hoppensteadt D., Bansal V., Singh A., Fareed J. (2013). Progressive increase of inflammatory biomarkers in chronic kidney disease and end-stage renal disease. Clin. Appl. Thromb. Hemost..

[134] Aydin Z., Gursu M., Karadag S., Uzun S., Sumnu A., Doventas Y., Ozturk S., Kazancioglu R. (2014). The relationship of prohepcidin levels with anemia and inflammatory markers in non-diabetic uremic patients: A controlled study. Ren. Fail..

[135] Goyal K.K., Saha A., Sahi P.K., Kaur M., Dubey N.K., Goyal P., Upadhayay A.D. (2018). Hepcidin and proinflammatory markers in children with chronic kidney disease: A case-control study. Clin. Nephrol..

[136] Musanovic A., Trnacevic S., Mekic M., Musanovic A. (2013). The influence of inflammatory markers and CRP predictive value in relation to the target hemoglobin level in patients on chronic hemodialysis. Med. Arch..

[137] Saltissi D., Sauvage D., Westhuyzen J. (1998). Comparative response to single or divided doses of parenteral iron for functional iron deficiency in hemodialysis patients receiving erythropoietin (EPO). Clin. Nephrol..

[138] Voulgarelis M., Kokori S.I., Ioannidis J.P., Tzioufas A.G., Kyriaki D., Moutsopoulos H.M. (2000). Anaemia in systemic lupus erythematosus: Aetiological profile and the role of erythropoietin. Ann. Rheum. Dis..

[139] Van Santen S., van Dongen-Lases E.C., de Vegt F., Laarakkers C.M., van Riel P.L., van Ede A.E., Swinkels D.W. (2011). Hepcidin and hemoglobin content parameters in the diagnosis of iron deficiency in rheumatoid arthritis patients with anemia. Arthritis Rheum..

[140] Seyhan S., Pamuk Ö.N., Pamuk G.E., Çakır N. (2014). The correlation between ferritin level and acute phase parameters in rheumatoid arthritis and systemic lupus erythematosus. Eur. J. Rheumatol..

[141] Cavallaro F., Duca L., Pisani L.F., Rigolini R., Spina L., Tontini G.E., Munizio N., Costa E., Cappellini M.D., Vecchi M. (2017). Anti-TNF-mediated modulation of prohepcidin improves iron availability in inflammatory bowel disease, in an IL-6-mediated fashion. Can. J. Gastroenterol. Hepatol..

[142] Stein J., Haas J.S., Ong S.H., Borchert K., Hardt T., Lechat E., Nip K., Foerster D., Braun S., Baumgart D.C. (2018). Oral versus intravenous iron therapy in patients with inflammatory bowel disease and iron deficiency with and without anemia in Germany—A real-world evidence analysis. Clinicoecon. Outcomes Res..

[143] Kronbichler A., Mayer G. (2013). Renal involvement in autoimmune connective tissue diseases. BMC Med..

[144] Ambruzs J.M., Walker P.D., Larsen C.P. (2014). The histopathologic spectrum of kidney biopsies in patients with inflammatory bowel disease. Clin. J. Am. Soc. Nephrol..

[145] Vanarsa K., Ye Y., Han J., Xie C., Mohan C., Wu T. (2012). Inflammation associated anemia and ferritin as disease markers in SLE. Arthritis Res. Ther..

[146] Tripathy R., Panda A.K., Das B.K. (2015). Serum ferritin level correlates with SLEDAI scores and renal involvement in SLE. Lupus.

[147] Umare V., Nadkarni A., Nadkar M., Rajadhyksha A., Khadilkar P., Ghosh K., Pradhan V.D. (2017). Do high sensitivity C-reactive protein and serum interleukin-6 levels correlate with disease activity in systemic lupus erythematosuspatients?. J. Postgrad. Med..

[148] Ripley B.J., Goncalves B., Isenberg D.A., Latchman D.S., Rahman A. (2005). Raised levels of interleukin 6 in systemic lupus erythematosus correlate with anaemia. Ann. Rheum. Dis..

[149] Mohammed M.F., Belal D., Bakry S., Marie M.A., Rashed L., Eldin R.E., El-Hamid S.A. (2014). A study of hepcidin and monocyte chemoattractant protein-1 in Egyptian females with systemic lupus erythematosus. J. Clin. Lab. Anal..

[150] Zhang X., Nagaraja H.N., Nadasdy T., Song H., McKinley A., Prosek J., Kamadana S., Rovin B.H. (2012). A composite urine biomarker reflects interstitial inflammation in lupus nephritis kidney biopsies. Kidney Int..

[151] Demirag M.D., Haznedaroglu S., Sancak B., Konca C., Gulbahar O., Ozturk M.A., Goker B. (2009). Circulating hepcidin in the crossroads of anemia and inflammation associated with rheumatoid arthritis. Intern. Med..

[152] Martinelli M., Strisciuglio C., Alessandrella A., Rossi F., Auricchio R., Campostrini N., Girelli D., Nobili B., Staiano A., Perrotta S. (2016). Serum hepcidin and iron absorption in paediatric inflammatory bowel disease. J. Crohns Colitis.

[153] Iqbal T., Stein J., Sharma N., Kulnigg-Dabsch S., Vel S., Gasche C. (2015). Clinical significance of C-reactive protein levels in predicting responsiveness to iron therapy in patients with inflammatory bowel disease and iron deficiency anemia. Dig. Dis. Sci..

[154] Ross D.N. (1950). Oral and intravenous iron therapy in the anaemia of rheumatoid arthritis. Ann. Rheum. Dis..

[155] Anuradha S., Singh N.P., Agarwal S.K. (2002). Total dose infusion iron dextran therapy in predialysis chronic renal failure patients. Ren. Fail..

[156] Rambod M., Kovesdy C.P., Kalantar-Zadeh K. (2008). Combined high serum ferritin and low iron saturation in hemodialysis patients: The role of inflammation. Clin. J. Am. Soc. Nephrol..

[157] Rafiean-Kopaie M., Nasri H. (2013). Impact of inflammation on anemia of hemodialysis patients who were under treatment of recombinant human erythropoietin. J. Ren. Inj. Prev..

[158] Tessitore N., Girelli D., Campostrini N., Bedogna V., Pietro Solero G., Castagna A., Melilli E., Mantovani W., De Matteis G., Olivieri O. (2010). Hepcidin is not useful as a biomarker for iron needs in haemodialysis patients on maintenance erythropoiesis-stimulating agents. Nephrol. Dial. Transplant..

[159] Gaillard C.A., Bock A.H., Carrera F., Eckardt K.U., Van Wyck D.B., Bansal S.S., Cronin M., Meier Y., Larroque S., Roger S.D. (2016). Hepcidin response to iron therapy in patients with non-dialysis dependent CKD: An analysis of the FIND-CKD trial. PLoS ONE.

[160] Yavuz A., Akbaş S.H., Tuncer M., Kolağasi O., Cetinkaya R., Gürkan A., Demirbaş A., Gultekin M., Akaydin M., Ersoy F. (2004). Influence of inflammation on the relation between markers of iron deficiency in renal replacement therapy. Transplant. Proc..

[161] Uehata T., Tomosugi N., Shoji T., Sakaguchi Y., Suzuki A., Kaneko T., Okada N., Yamamoto R., Nagasawa Y., Kato K. (2012). Serum hepcidin-25 levels and anemia in non-dialysis chronic kidney disease patients: A cross-sectional study. Nephrol. Dial. Transplant..

[162] Chand S., Ward D.G., Ng Z.Y., Hodson J., Kirby H., Steele P., Rooplal I., Bantugon F., Iqbal T., Tselepis C. (2015). Serum hepcidin-25 and response to intravenous iron in patients with non-dialysis chronic kidney disease. J. Nephrol..

[163] Drakou A., Margeli A., Theodorakopoulou S., Agrogiannis I., Poziopoulos C., Papassotiriou I., Vlahakos D.V. (2016). Assessment of serum bioactive hepcidin-25, soluble transferrin receptor and their ratio in predialysis patients: Correlation with the response to intravenous ferric carboxymaltose. Blood Cells Mol. Dis..

[164] Lenga I., Lok C., Marticorena R., Hunter J., Dacouris N., Goldstein M. (2007). Role of oral iron in the management of long-term hemodialysis patients. Clin. J. Am. Soc. Nephrol..

[165] Tsuchida A., Paudyal B., Paudyal P., Ishii Y., Hiromura K., Nojima Y., Komai M. (2010). Effectiveness of oral iron to manage anemia in long-term hemodialysis patients with the use of ultrapure dialysate. Exp. Ther. Med..

[166] Nagaraju S.P., Cohn A., Akbari A., Davis J.L., Zimmerman D.L. (2013). Heme iron polypeptide for the treatment of iron deficiency anemia in non-dialysis chronic kidney disease patients: A randomized controlled trial. BMC Nephrol..

[167] Bhandari S., Kalra P.A., Kothari J., Ambühl P.M., Christensen J.H., Essaian A.M., Thomsen L.L., Macdougall I.C., Coyne D.W. (2015). A randomized, open-label trial of iron isomaltoside 1000 (Monofer^®^) compared with iron sucrose (Venofer^®^) as maintenance therapy in haemodialysis patients. Nephrol. Dial. Transplant..

[168] Rostoker G., Griuncelli M., Loridon C., Magna T., Machado G., Drahi G., Dahan H., Janklewicz P., Cohen Y. (2015). Reassessment of iron biomarkers for prediction of dialysis iron overload: An MRI study. PLoS ONE.

[169] Tanaka A., Inaguma D., Watanabe Y., Ito E., Kamegai N., Shimogushi H., Shinjo H., Koike K., Otsuka Y., Takeda A. (2017). Ferrokinetics is associated with the left ventricular mass index in patients with chronic kidney disease. Acta Cardiol..

[170] Lin K.C., Tsai M.Y., Chi C.L., Yu L.K., Huang L.H., Chen C.A. (2015). Serum ferritin is associated with arterial stiffness in hemodialysis patients: Results of a 3-year follow-up study. Int. Urol. Nephrol..

[171] Reis K.A., Guz G., Ozdemir H., Erten Y., Atalay V., Bicik Z., Ozkurt Z.N., Bali M., Sindel S. (2005). Intravenous iron therapy as a possible risk factor for atherosclerosis in end-stage renal disease. Int. Heart J..

[172] Hsieh Y.P., Huang C.H., Lee C.Y., Chen H.L., Lin C.Y., Chang C.C. (2013). Hepcidin-25 negatively predicts left ventricular mass index in chronic kidney disease patients. World J. Nephrol..

[173] Kuragano T., Itoh K., Shimonaka Y., Kida A., Furuta M., Kitamura R., Yahiro M., Nanami M., Otaki Y., Hasuike Y. (2011). Hepcidin as well as TNF-α are significant predictors of arterial stiffness in patients on maintenance hemodialysis. Nephrol. Dial. Transplant..

[174] Li H., Feng S.J., Su L.L., Wang W., Zhang X.D., Wang S.X. (2015). Serum hepcidin predicts uremic accelerated atherosclerosis in chronic hemodialysis patients with diabetic nephropathy. Chin. Med. J. (Engl.).

[175] Mostovaya I.M., Bots M.L., van den Dorpel M.A., Goldschmeding R., den Hoedt C.H., Kamp O., Levesque R., Mazairac A.H., Penne E.L., Swinkels D.W. (2014). Left ventricular mass in dialysis patients, determinants and relation with outcome. Results from the COnvective TRansport STudy (CONTRAST). PLoS ONE.

[176] Van der Weerd N.C., Grooteman M.P., Bots M.L., van den Dorpel M.A., den Hoedt C.H., Mazairac A.H., Nubé M.J., Penne E.L., Wetzels J.F., Wiegerinck E.T. (2013). Hepcidin-25 is related to cardiovascular events in chronic haemodialysis patients. Nephrol. Dial. Transplant..

[177] Rostoker G., Hummel A., Chantrel F., Ryckelynck J.P. (2014). Therapy of anemia and iron deficiency in dialysis patients: An update. Nephrol. Ther..

[178] Del Vecchio L., Locatelli F. (2017). Clinical practice guidelines on iron therapy: A critical evaluation. Hemodial. Int..

[179] Al-Hawas F., Abdalla A.H., Popovich W., Mousa D.H., al-Sulaiman M.H., al-Khader A.A. (1997). Use of i.v. iron saccharate in haemodialysis patients not responding to oral iron and erythropoietin. Nephrol. Dial. Transplant..

[180] Canavese C., Bergamo D., Ciccone G., Burdese M., Maddalena E., Barbieri S., Thea A., Fop F. (2004). Low-dose continuous iron therapy leads to a positive iron balance and decreased serum transferrin levels in chronic haemodialysis patients. Nephrol. Dial. Transplant..

[181] Rostoker G., Griuncelli M., Loridon C., Magna T., Janklewicz P., Drahi G., Dahan H., Cohen Y. (2014). Maximal standard dose of parenteral iron for hemodialysis patients: An MRI-based decision tree learning analysis. PLoS ONE.

[182] Vaziri N.D. (2016). Safety issues in iron treatment in CKD. Semin. Nephrol..

[183] Locatelli F., Mazzaferro S., Yee J. (2016). Iron therapy challenges for the treatment of nondialysis CKD patients. Clin. J. Am. Soc. Nephrol..

[184] Agarwal R., Kusek J.W., Pappas M.K. (2015). A randomized trial of intravenous and oral iron in chronic kidney disease. Kidney Int..

[185] Litton E., Xiao J., Ho K.M. (2013). Safety and efficacy of intravenous iron therapy in reducing requirement for allogeneic blood transfusion: Systematic review and meta-analysis of randomised clinical trials. BMJ.

[186] Bailie G.R., Larkina M., Goodkin D.A., Li Y., Pisoni R.L., Bieber B., Mason N., Tong L., Locatelli F., Marshall M.R. (2015). Data from the Dialysis Outcomes and Practice Patterns Study validate an association between high intravenous iron doses and mortality. Kidney Int..

[187] Miskulin D.C., Tangri N., Bandeen-Roche K., Zhou J., McDermott A., Meyer K.B., Ephraim P.L., Michels W.M., Jaar B.G., Crews D.C. (2014). Developing Evidence to Inform Decisions about Effectiveness (DEcIDE) Network Patient Outcomes in End Stage Renal Disease Study Investigators. Intravenous iron exposure and mortality in patients on hemodialysis. Clin. J. Am. Soc. Nephrol..

[188] Drüeke T., Witko-Sarsat V., Massy Z., Descamps-Latscha B., Guerin A.P., Marchais S.J., Gausson V., London G.M. (2002). Iron therapy, advanced oxidation protein products, and carotid artery intima-media thickness in end-stage renal disease. Circulation.

[189] Kim S.M., Lee C.H., Oh Y.K., Joo K.W., Kim Y.S., Kim S., Lim C.S. (2011). The effects of oral iron supplementation on the progression of anemia and renal dysfunction in patients with chronic kidney disease. Clin. Nephrol..

[190] Agarwal R., Rizkala A.R., Kaskas M.O., Minasian R., Trout J.R. (2007). Iron sucrose causes greater proteinuria than ferric gluconate in non-dialysis chronic kidney disease. Kidney Int..

[191] Maruyama Y., Nakayama M., Yoshimura K., Nakano H., Yamamoto H., Yokoyama K., Lindholm B. (2007). Effect of repeated intravenous iron administration in haemodialysis patients on serum 8-hydroxy-2′-deoxyguanosine levels. Nephrol. Dial. Transplant..

[192] Yahiro M., Kuragano T., Kida A., Kitamura R., Furuta M., Hasuike Y., Otaki Y., Nonoguchi H., Nakanishi T. (2012). The impact of ferritin fluctuations on stable hemoglobin levels in hemodialysis patients. Clin. Exp. Nephrol..

[193] Wish J.B. (2011). Anemia management under a bundled payment policy for dialysis: A preview for the United States from Japan. Kidney Int..

[194] Kato S., Lindholm B., Yuzawa Y., Tsuruta Y., Nakauchi K., Yasuda K., Sugiura S., Morozumi K., Tsuboi N., Maruyama S. (2016). High ferritin level and malnutrition predict high risk of infection-related hospitalization in incident dialysis patients: A Japanese prospective cohort study. Blood Purif..

[195] Ramanathan G., Olynyk J.K., Ferrari P. (2017). Diagnosing and preventing iron overload. Hemodial. Int..

[196] Canavese C., Bergamo D., Ciccone G., Longo F., Fop F., Thea A., Martina G., Piga A. (2004). Validation of serum ferritin values by magnetic susceptometry in predicting iron overload in dialysis patients. Kidney Int..

[197] Ghoti H., Rachmilewitz E.A., Simon-Lopez R., Gaber R., Katzir Z., Konen E., Kushnir T., Girelli D., Campostrini N., Fibach E. (2012). Evidence for tissue iron overload in long-term hemodialysis patients and the impact of withdrawing parenteral iron. Eur. J. Haematol..

[198] Vaziri N.D. (2012). Epidemic of iron overload in dialysis population caused by intravenous iron products: A plea for moderation. Am. J. Med..

[199] Nissenson A.R., Berns J.S., Sakiewicz P., Ghaddar S., Moore G.M., Schleicher R.B., Seligman P.A. (2003). Clinical evaluation of heme iron polypeptide: Sustaining a response to rHuEPO in hemodialysis patients. Am. J. Kidney Dis..

[200] Tanemoto M., Ishimoto Y., Saito H. (2017). Oral low-dose ferric citrate is a useful iron source for hyperphosphatemic hemodialysis patients: A case series. Blood Purif..

[201] Pandey R., Daloul R., Coyne D.W. (2016). Iron treatment strategies in dialysis-dependent CKD. Semin. Nephrol..

[202] Malovrh M., Hojs N., Premru V. (2011). The influence of need-based, continuous, low-dose iron replacement on hemoglobin levels in hemodialysis patients treated with erythropoiesis-stimulating agents. Artif. Organs.

[203] Deira J., González-Sanchidrián S., Polanco S., Cebrián C., Jiménez M., Marín J., Gómez-Martino J.R., Fernández-Pereira L., Tabernero J. (2016). Very low doses of direct intravenous iron in each session as maintenance therapy in hemodialysis patients. Ren. Fail..

[204] Schiesser D., Binet I., Tsinalis D., Dickenmann M., Keusch G., Schmidli M., Ambühl P.M., Lüthi L., Wüthrich R.P. (2006). Weekly low-dose treatment with intravenous iron sucrose maintains iron status and decreases epoetin requirement in iron-replete haemodialysis patients. Nephrol. Dial. Transplant..

[205] Strauss W.E., Dahl N.V., Li Z., Lau G., Allen L.F. (2016). Ferumoxytol versus iron sucrose treatment: A post-hoc analysis of randomized controlled trials in patients with varying renal function and iron deficiency anemia. BMC Hematol..

[206] Rath T., Florschütz K., Kalb K., Rothenpieler U., Schletter J., Seeger W., Zinn S. (2010). Low-molecular-weight iron dextran in the management of renal anaemia in patients on haemodialysis-the IDIRA Study. Nephron Clin. Pract..

[207] Gupta A., Lin V., Guss C., Pratt R., Ikizler T.A., Besarab A. (2015). Ferric pyrophosphate citrate administered via dialysate reduces erythropoiesis-stimulating agent use and maintains hemoglobin in hemodialysis patients. Kidney Int..

[208] Ferrari P., Mallon D., Trinder D., Olynyk J.K. (2010). Pentoxifylline improves haemoglobin and interleukin-6 levels in chronic kidney disease. Nephrology (Carlton).

[209] Pergola P.E., Spinowitz B.S., Hartman C.S., Maroni B.J., Haase V.H. (2016). Vadadustat, a novel oral HIF stabilizer, provides effective anemia treatment in nondialysis-dependent chronic kidney disease. Kidney Int..

[210] Martin E.R., Smith M.T., Maroni B.J., Zuraw Q.C., deGoma E.M. (2017). Clinical trial of vadadustat in patients with anemia secondary to stage 3 or 4 chronic kidney disease. Am. J. Nephrol..

[211] Besarab A., Provenzano R., Hertel J., Zabaneh R., Klaus S.J., Lee T., Leong R., Hemmerich S., Yu K.H., Neff T.B. (2015). Randomized placebo-controlled dose-ranging and pharmacodynamics study of roxadustat (FG-4592) to treat anemia in nondialysis-dependent chronic kidney disease (NDD-CKD) patients. Nephrol. Dial. Transplant..

[212] Besarab A., Chernyavskaya E., Motylev I., Shutov E., Kumbar L.M., Gurevich K., Chan D.T., Leong R., Poole L., Zhong M. (2016). Roxadustat (FG-4592): Correction of anemia in incident dialysis patients. J. Am. Soc. Nephrol..

[213] Brigandi R.A., Johnson B., Oei C., Westerman M., Olbina G., de Zoysa J., Roger S.D., Sahay M., Cross N., McMahon L. (2016). A novel hypoxia-inducible factor-prolyl hydroxylase inhibitor (GSK1278863) for anemia in CKD: A 28-day, phase 2a randomized trial. Am. J. Kidney Dis..

[214] Kaysen G.A. (2009). Biochemistry and biomarkers of inflamed patients: Why look, what to assess. Clin. J. Am. Soc. Nephrol..

[215] Hsu P.Y., Lin C.L., Yu C.C., Chien C.C., Hsiau T.G., Sun T.H., Huang L.M., Yang C.W. (2004). Ultrapure dialysate improves iron utilization and erythropoietin response in chronic hemodialysis patients—A prospective cross-over study. J. Nephrol..

[216] Sarafidis P.A., Rumjon A., MacLaughlin H.L., Macdougall I.C. (2012). Obesity and iron deficiency in chronic kidney disease: The putative role of hepcidin. Nephrol. Dial. Transplant..

[217] Sanad M., Osman M., Gharib A. (2011). Obesity modulate serum hepcidin and treatment outcome of iron deficiency anemia in children: A case control study. Ital. J. Pediatr..

